# Non-specialist delivered psycho-social interventions for women with perinatal depression living in rural communities: A systematic review

**DOI:** 10.1371/journal.pgph.0003031

**Published:** 2024-07-08

**Authors:** Anouk Ackerman, Nimrah Afzal, Alexandra Lautarescu, Claire A. Wilson, Abhijit Nadkarni

**Affiliations:** 1 Department of Population Health, London School of Hygiene and Tropical Medicine, London, United Kingdom; 2 UCLA David Geffen School of Medicine, Los Angeles, California, United States of America; 3 Department of Psychology, University of Bath, Bath, United Kingdom; 4 Department of Health Service and Population Research, Institute of Psychiatry, Psychology and Neuroscience, King’s College London, London, United Kingdom; 5 South London and Maudsley NHS Foundation Trust, London, United Kingdom; Institute of Tropical Medicine: Instituut voor Tropische Geneeskunde, BELGIUM

## Abstract

Evidence from low- and middle-income countries suggests that non-specialist-delivered interventions effectively improve access to perinatal mental health care. However, there have been no systematic attempts to synthesize the evidence on effectiveness, relevance, and application of this strategy to resource-limited settings such as rural areas. The aim of this review is to synthesize the evidence about the effectiveness of non-specialist delivered interventions in improving depression and related outcomes in women with perinatal depression living in rural communities. Seven electronic databases were searched using the following search concepts: perinatal depression (e.g., puerperal depression, antenatal depression), rural areas (e.g., remote, nonmetropolitan, underserved), and non-specialist workers (e.g., lay worker, volunteer aide, informal caretaker. The risk of bias was assessed using RoB-2 and ROBINS-I tools. A narrative synthesis was performed as the high degree of study heterogeneity precluded a meta-analysis. Nine unique studies were eligible for inclusion. Psychoeducation and problem-solving techniques were the most used intervention elements. Two interventions significantly reduced the prevalence of perinatal depression compared to usual care, and three interventions reported effectiveness in reducing depression symptom severity. There was little to no consistent evidence for other outcomes, including but not limited to maternal health care utilization, breastfeeding behaviors, and child health. This review provides limited evidence to suggest that non-specialist delivered interventions effectively improved outcomes among women with perinatal depression living in rural communities. The paucity of high-quality studies included in this review demonstrates that this rural demographic is frequently neglected in the context of maternal mental health research.

## Introduction

Perinatal depression (PD), defined as depression occurring during pregnancy or within 12 months of delivery, is one of the most common complications of pregnancy worldwide [1]. Globally, 10–25% of women may experience PD, with women in low-resource settings being disproportionately affected [2, 3].

Untreated PD has been associated with numerous adverse outcomes for mothers and their offspring. For example, children may experience stunted postnatal growth or increased risk of later mental health difficulties, while mothers may be at higher risk for obstetric complications or premature delivery [4]. Although less frequent, infanticide or maternal suicide may also occur in severe cases [5, 6].

Treatment for PD frequently involves individual or group psychotherapy, although psychotropic medications such as antidepressants may also be used [7, 8]. Several psychological and psychosocial approaches, such as interpersonal therapy (IPT), cognitive behavioral therapy (CBT), and nondirective counselling, have been reported to be effective [9, 10]. Despite well-established, effective treatment modalities, only a small minority of women receive adequate perinatal mental health care. For example, only 10–15% of women living in high-income countries (HICs) who meet diagnostic criteria for PD receive comprehensive treatment [11]. This proportion is likely much lower in resource-constrained settings where mental health specialists are scarce and access to quality care remains limited [12].

Interventions delivered by non-specialists (also known as task sharing) have been widely recognized as an effective means of improving access to mental health care [12–14]. This strategy has been applied successfully to the provision of maternal mental health care worldwide. Non-specialist-delivered psychological interventions produced an overall reduction in common perinatal mental disorders symptomatology compared to usual perinatal care when delivered to women in low-income countries (LMICs) (SMD -0.34; 95% CI -0.53, -0.16) [15]; similar findings have also been reported in HICs [14].

While particular attention has been paid to the use of non-specialists for maternal mental health in LMICs, our understanding of their relevance and application in low-resource settings must also include rural communities. Regardless of country income status, rural communities worldwide suffer disproportionately from unemployment, poverty, and poor educational opportunities compared to urban areas [16–18]. Limited access to basic public health services, including mental health care, further compounds this urban-rural disparity [19, 20]. Non-specialists offer a potential solution to address this inequity; however, rural areas present unique challenges to the provision of mental health care, and strategies effective in urban areas (regardless of country income status) cannot be assumed to reliably apply to a rural context.

This review seeks to address the notable research gap related to utilizing non-specialists to deliver maternal mental health care in rural communities. Its goal is to enhance our understanding of the most effective and sustainable methods of mental health care delivery in this underserved and in-need population.

The specific objectives of this review are as follows:

1. To assess the effectiveness of non-specialist delivered interventions in improving outcomes related to depression including but not limited to depressive symptom severity and remission in women with perinatal depression living in rural communities;
2. To assess the effectiveness of such interventions in improving infant wellbeing and related outcomes including but not limited to physical health and cognitive development; and
3. To examine the content, processes, and elements of these interventions to aid in the design and implementation of future maternal mental health programs.

## Methods

This systematic review was registered on PROSPERO on January 18^th^, 2023 (CRD42023387400) and reported as per the Preferred Reporting Items for Systematic Reviews and Meta-analyses (PRISMA).

### Eligibility criteria

Eligible studies included female participants of any age experiencing perinatal depression, defined as during pregnancy or within 12 months of delivery and confirmed through standardized screening tools and/or diagnostic interviews. Participants were included regardless of any other co-occurring mental health conditions like anxiety. Any intervention with a non-pharmacological component (including combined psychosocial and pharmacological approaches), delivered by non-specialist workers (NSW) was considered eligible provided one or more depression-related outcomes (e.g., symptom severity, remission) were measured as either a primary or secondary outcome. NSW were defined as anyone without specialized mental health qualifications (e.g., primary care physicians, nurses, midwives, lay persons, health workers/volunteers, peers). Randomized controlled trials or quasi-experimental studies that compared the intervention to any of the following: treatment as usual, no perinatal care, placebo, or other interventions (including pharmacological ones) met study design criteria. Studies conducted in a rural setting(s) worldwide were included. Due to the heterogeneity in the definition of “rural”, a study was included if the authors defined the setting as rural.

### Search strategy

Search terms were organized under each of the following concepts: perinatal depression (e.g., puerperal depression, antenatal depression), rural areas (e.g., remote, nonmetropolitan, underserved), and non-specialist workers (e.g., lay worker, volunteer aide, informal caretaker). Two edits were made post protocol publication to increase search sensitivity. First, the search term “pregnancy” was changed to “pregnan*” to include variations such as “pregnant” and “pregnancies”. Second, the broader search term “disorder*” was used, as it was more inclusive than “common mental disorder” and/or “affective mental disorder” alone. Detailed search strategies are presented in the Supplementary Materials (Table D in S1 Text).

Seven databases (Cumulative Index to Nursing and Allied Health Literature, MEDLINE, EMBASE, PsycINFO, Global Health, Cochrane CENTRAL, Global Index Medicus) were searched from their inception to January 2023. The only restriction applied was for English language in Global Index Medicus due to the preponderance of non-English results with no available translation. Records were exported to Zotero before de-duplication using the Rayyan systematic review management software. All flagged duplicates were manually verified for accuracy.

Additional methods to identify potentially eligible studies included a) searching reference lists of relevant systematic reviews; b) forward and backward citation chaining of eligible papers; and c) hand-searching reference lists of eligible papers. Finally, five independent experts in maternal mental health (Table E in S1 Text) were contacted to identify any eligible studies that may have been missed.

### Selection of studies

Assessment for inclusion was a two-stage screening process performed independently by two reviewers (AA, NA) using the Rayyan software. Both reviewers screened 100% of the records (stage 1 and stage 2) and the “blind” functionality was used to maintain objectivity.

During stage 1, both reviewers independently screened the title and abstract. The blind function was turned off when 10% of articles were screened to ensure a mutual understanding of inclusion criteria. Conflicts were resolved through discussion, and the blind function was then reactivated until all titles and abstracts were screened. If an abstract did not provide enough information to determine eligibility, the full text was reviewed. For records with unavailable full texts, corresponding authors (n = 5) were contacted, four of whom responded. Those four studies were not eligible as the authors reported that the corresponding study results were either incomplete or unpublished. After stage 1, the blind function was again turned off, and conflicts (4%) were resolved through discussion without the need for a third reviewer.

In stage 2, both reviewers independently screened 100% of the articles meeting the criteria for full-text review. Again, the blind function was turned off after screening 8–10% of articles to ensure mutual understanding of inclusion and exclusion criteria. The blind function was then reactivated until all full texts were screened. Subsequently, conflicts (21%) were discussed with the blind function off and resolved without the need for a third party.

When information required to determine whether a study met eligibility criteria was missing, the first reviewer (AA) contacted the study authors to obtain the necessary information, and the study was excluded if the authors were unresponsive even after three attempts to contact them. Authors of studies that reported including participants in rural and urban locations were contacted to obtain results for the rural sub-sample (if available). The registration pages of trial protocols identified during the search were also searched for completed trials and/or relevant publications.

### Data extraction and management

Using an Excel data extraction form informed by the Cochrane data extraction form and customized to reflect study aims, both reviewers (AA, NA) independently conducted data extraction (Table C in S1 Text). Extracted data for each study included country, intervention setting, method and agent of delivery, sample size, participant demographics, baseline characteristics, intervention details, study design, outcomes, and measurement timing/tools (e.g., depression measurement method). Discrepancies in data entry between the two reviewers were resolved through discussion and joint review of published data in the relevant paper.

### Assessment of risk of bias

Two reviewers (AA, NA) independently performed bias assessments. The Risk Of Bias In Non-Randomized Studies of Interventions (ROBINS-I) tool [21] was used for non-randomized study designs and the Risk of Bias-2 (RoB-2) tool [22] for randomized controlled trials (RCTs).

RCTs were evaluated based on RoB2 domains, including random sequence generation, allocation concealment, blinding, incomplete outcome data, selective reporting, and others. Non-randomized studies were assessed for bias related to confounding, participant selection, intervention classification, deviations, missing data, outcome measurement, and result reporting. Studies were classified as having low, medium, or high risk of bias. Disagreements were resolved through discussion.

### Analysis

Both reviewers (AA, NA) subsequently conducted a narrative synthesis of the data. Mean values for primary endpoints and corresponding effect sizes were extracted for each study; however, the significant heterogeneity between studies, encompassing factors such as the primary techniques utilized (e.g., cognitive restructuring, problem-solving, child education), the mode and means of delivery (e.g., group or individual sessions and various types of non-specialists), and the intensity (e.g., session count, duration, and frequency) for each intervention precluded a meta-analysis.

Following Cochrane Collaboration recommendations [23], reviewers organized study results in tables to identify patterns, explored differences between effective and non-effective interventions, and tabulated factors explaining variations. Interventions were clustered based on components to identify patterns within or across groups. Salient patterns and themes were textually described.

## Results

**Fig 1:**
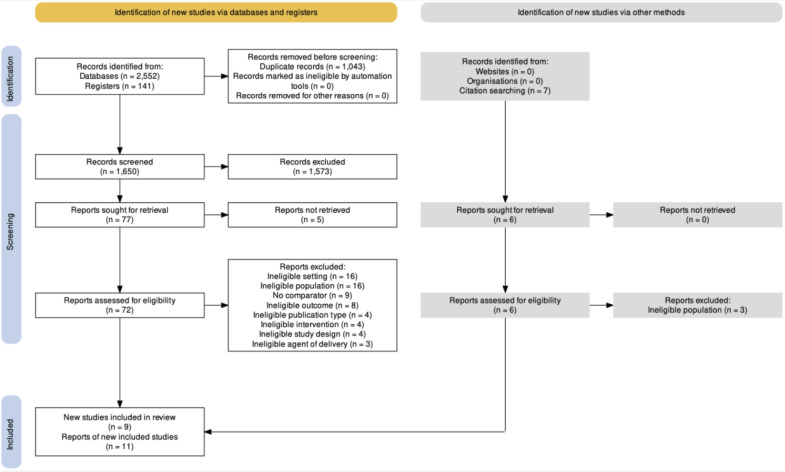
PRISMA diagram. NOTE: Seven additional records were identified via citation searching. Of those seven, reviewers (AA, NA) agreed that one did not pass title and abstract screening. As such, only six full-text records were sought for retrieval.

Following PRISMA guidelines, the entire screening process is summarized (Fig 1). Eleven reports representing nine unique studies were eligible for inclusion in this review. One record [24] examined 12-month outcomes among the same group of participants who had received the original Philani Mentor Mother intervention [25]. Similarly, Maselko and colleagues examined outcomes 36 months later in the same cohort of women who had initially received the Thinking Healthy Peer-Delivered Program reported in another record [26].

### Study characteristics (Table 1)

**Table 1:** Study characteristics.

Author	Country	Intervention Setting	Design	Stage of Antenatal Period at Enrolment	Baseline Maternal Depression Eligibility Criteria	Age (years)		Intervention, *n*	Control, *n*
						Mean (SD)	Age Range		
Alfayumi-Zeadna et al., 2022 [27]	Israel	Women’s health clinics	Non-randomized controlled trial	26 – 38w pregnant	None	28.5 (6.1)	16–40	Clinic-based psychoeducation, *n =* 169	Standard antenatal care, *n =* 163
Comrie-Thomson et al., 2022 [28]	Zimbabwe	Communal location	Cluster-RCT	0–6 months postpartum	None	25.1 (6.6) [I]; 25.8 (6.7) [C]	≥16	Mbereko and Men: gender-synchronized, community-based problem-solving and psychoeducation, *n =* 436	Standard antenatal care, *n =* 454
George et al., 2020 [33]	India	Communal location	Non-randomized controlled trial	<32w pregnant	None	25.0 (3.21)	NR	Group-based problem-solving with components of cognitive behavioral therapy, *n =* 69	Active control—psychoeducation session on pregnancy, breast feeding, immunization, *n = 75* Negative control—standard antenatal care, *n =* 70
Khan et al., 2017 [30]	Pakistan	Participant home	RCT (feasibility trial)	Pregnant (no trimester criteria)	SRQ ≥ 9	[I]/[C] < = 19yo = 12% / 16%, 20–24 = 24% / 32%, 25–29 = 35% / 27%, 30+ = 29% / 24%		Happy Mother, Healthy Child in Ten Steps: family-based psychoeducation, *n =* 34	Standard antenatal care, *n =* 39
Le Roux et al., 2020 [25]	South Africa	Participant home	Non-randomized controlled trial	Pregnant (no trimester criteria)	None	NR	14–54 (median 24)	Mentor Mother Community Health Worker Program: individual psycho- and child health education, *n =* 636	Standard antenatal care, *n =* 674
Maselko et al., 2020 [31]	Pakistan	Participant home, communal location	Cluster-RCT	>24w pregnant	PHQ-9 ≥10	26.7 (4.5)	≥18	Thinking Healthy Program Peer-Delivered Plus, THPP+: group and individual cognitive behavioral therapy-based sessions, *n =* 283	Enhanced antenatal care^s^, *n =* 287
Rahman A. et al., 2008 [32]	Pakistan	Participant home	Cluster-RCT	>24w pregnant	Must meet DSM-IV criteria for perinatal depression	26.5 (5.2) [I], 27 (5.1) [C]	16–45	Thinking Healthy Program, THP: individual sessions based on techniques of cognitive behavioral therapy, *n =* 463	Enhanced antenatal care^ss^, *n =* 440
Sikander et al., 2019 [26]	Pakistan	Participant home, health house or other communal location	Cluster-RCT	>24w pregnant	PHQ-9 ≥10	26.8 (4.6) [I], 27.28 (4.97) [C]	≥ 18	Thinking Healthy Program Peer-Delivered, THPP: group and individual cognitive behavioral therapy-based sessions, *n =* 283	Enhanced antenatal care^s^, *n =* 287
Stansert Katzen et al., 2020 [29]	South Africa	Participant home	Non-randomized cohort study	0 – 2w postpartum	None	24.8 (6.3) [I], 24.9 (7.6) [C]	NR	Mentor Mother Community Health Worker Program: individual psychoeducation, child health education, *n =* 157	Standard antenatal and HIV care, *n =* 313
Stansert Katzen et al., 2021 [24]	South Africa	Participant home	Non-randomized cohort study	Pregnant (no trimester criteria)	None	NR	15–54 (median 24)	Mentor Mother Community Health Worker Program: individual psychoeducation, child health education, *n =* 636	Standard antenatal and primary health care, *n =* 674
Tripathy et al., 2010 [34]	India	Communal location	Cluster-RCT	Postpartum (gave birth during study, 2005–2008)	None	[I]/[C] <20 = 6% / 11% 20–29 = 56% / 62% >30 = 14% / 16% Unknown = 38% / 26%		Participatory women’s groups, *n =* 2457	Standard antenatal care, *n =* 2235

[C]–control, [I]–intervention, [I]/[C]–intervention/control, NR–not reported, RCT–randomized control trial, SD–standard deviation, W–weeks. Gestational age was calculated from the first day of last menstrual period.

NOTE: Three studies described the control group as receiving enhanced antenatal care [26, 31, 32]. In the study performed by Rahman and colleagues, enhanced antenatal care involved control participants receiving an equal number of home visits as the intervention group, although visits were by a CHW not trained in the intervention. Conversely, control participants in the other records were provided standard antenatal care in addition to being informed of their depression screening results and how to seek the appropriate care.

Apart from a solitary study conducted in Israel (n = 1) [27], the remaining studies were conducted in LMICs including Zimbabwe (n = 1) [28], South Africa (n = 2) [24, 25, 29], Pakistan (n = 3) [26, 30–32], and India (n = 2) [33, 34].

Five studies were RCTs [26, 28, 30–32, 34] (four of which were cluster-RCTs) and the remaining were quasi-experimental in design [24, 25, 27, 29, 33].

Five studies included adolescent participants (<18) or did not report age exclusion criteria [24, 25, 27, 28, 32, 34]. Six studies enrolled pregnant women [24–27, 30–33] and the rest enrolled participants postpartum, all within less than six months of delivery [28, 29, 34]. One study included women and their male partners as participants [28].

Three studies required all participants meet specified criteria for perinatal depression (e.g., Edinburgh Postnatal Depression Score (EPDS) ≥ 13) for enrolment [26, 30–32]. Although other studies included participants not considered to have or be at high risk for perinatal depression, they provided stratified results for a subgroup meeting such criteria, making them eligible for inclusion.

### Interventions (Tables 1 and 2)

**Table 2:** Intervention characteristics.

Author	Primary Strategies*						Materials Used	Individual vs. Group	Primary Agent of Delivery (AoD)	Duration of AoD Training	Supervision of AoD	Method of Delivery	Number of Sessions (total)	Duration of Session (min)	Freq of Sessions
	Psychoeducation	Cognitive restructuring	Problem-Solving	Child Health Education	Psychosocial Support	Behavioral Activation									
Alfayumi-Zeadna et al., 2022 [27]	X						NR	Both	Nurses, gynacologists	“One session”	NR	FTF	≥3	NR	NR, delivered over 5 months
Comrie-Thomson et al., 2022 [28]	X		X		X		Flip charts, Action Birth Cards, Men’s Chart for Family Health	Group	CHW	NR	Trained female project staff member supervised all CHW-led sessions	FTF	12–15	60	Monthly
George et al., 2020 [33]		X	X				Informational handouts	Group	CHW	30–50 hours	NR	FTF	9	45–60	3x/day
Khan et al., 2017 [30]	X			X	X		Informational handouts, counselling cards	Group (family-based)	CHW	NR	Half of all sessions observed by supervisor with checklist	FTF	2	20–60	NR, delivered over 2 months
Le Roux et al., 2020 [25]	X			X			NR	Individual	CHW	6 weeks	“Daily in-the-field support and supervision visits”	FTF	>20**	NR	Weekly–monthly
Maselko et al., 2020 [31]	X		X		X	X	NR	Both	Volunteer peers	“Brief classroom training with regular group training and field supervision"	Monthly supervision with competency checklist and feedback; direct observation by peers	FTF	32	30–45	Monthly—bi-monthly
Rahman A. et al., 2008 [32]	X	X	X	X		X	Health calendar	Individual	CHW	2 days	Monthly half-day group supervision by research team members	FTF	16	45	Weekly–monthly
Sikander et al., 2019 [26]	X	X	X	X		X	NR	Both	Volunteer peers	“Brief classroom training with regular group training"	“Regular” field supervision by local THPP trainers	FTF	14	30–45	Weekly–monthly
Stansert Katzen et al., 2020 [29]	X			X			NR	Individual	CHW	6 weeks	“Supportive in-the-field supervision”	FTF	11	NR	Weekly–monthly
Stansert Katzen et al., 2021 [24]	X			X			NR	Individual	CHW	6 weeks	“Daily in-the-field support visits by supervisors”	FTF	>20**	NR	Weekly–monthly
Tripathy et al., 2010 [34]	X		X	X			Picture-card games	Group	Volunteer peers	1 week	“Fortnightly meetings with district coordinators”	FTF	20	NR	Monthly

CHW–community health workers, FTF = face-to-face, NR–not reported.

* Based on the primary intervention strategies as conceptualized by [36].

** Every month early in pregnancy, twice in the eighth month of pregnancy, weekly in the ninth month of pregnancy, within 2–3 days of arriving home after birth, weekly in the first month after birth and then every 2 weeks up until the infant is 1 year old. Monthly thereafter after.

In five studies at least part, if not all, of the intervention was delivered at the participants’ homes [24–26, 29–32]. One intervention was delivered at a primary care setting [27], while others were delivered at an unspecified communal location or the homes of the community health workers [28, 33, 34].

Three studies delivered some form of the Thinking Healthy Program [26, 31, 32], a World Health Organization (WHO) endorsed intervention that has been adapted for use in numerous contexts [35]. Another three studies delivered the Philani Mentor Mother Program [24, 25, 29], and the remaining studies delivered interventions designed specifically for each program.

All interventions aimed to benefit the mother, the mother-child dyad, or both and utilized strategies such as psychoeducation, problem-solving therapy, behavioral activation, or psychosocial support. Psychoeducation, which most frequently involved providing information about perinatal depression and general maternal and child health, was the most common strategy. Some form of problem-solving therapy was also utilized as a key intervention strategy in many studies [26, 28, 31–34]. Behavioral activation and psychosocial support were particularly relevant in interventions for which peers served as the primary agent of delivery [26, 31, 34].

All interventions were delivered face-to-face. Interventions were delivered in a group format (n = 4), [28, 30, 33, 34], an individual format (n = 3) [24, 25, 29, 32], or some combination of individual and group sessions (n = 2) [26, 27, 31].

Delivery agents included community health workers (CHW) [24, 25, 28–30, 32, 33], female peers or community members [26, 31, 34], and female clinic staff (e.g., nurses, gynecologists) [27], and the extent of training received by each delivery agent varied significantly.

The number, duration, and frequency of sessions were also highly variable. Session duration ranged from 20 minutes [30] to 1 hour [28], and the number of individual intervention sessions ranged from three [27] to thirty-two [31]. Two studies did not explicitly mention the number of sessions received by participants [24, 25, 29]. The most intense frequency included multiple individual sessions within one day [33], while the longest time between sessions was every other month [31].

### Study outcomes (Tables 1 and 3)

**Table 3:** Study outcomes.

Author	Assessment End Point	Maternal-Outcome(s) (as measured by)	Main Results (95% CI)	Infant Outcome(s) (as measured by)	Main Results (95% CI)
Alfayumi-Zeadna et al., 2022 [27]	21 weeks post-intervention	Prevalence of depressed mood^1^ (EPDS ≥ 10)	Greater decrease in proportion of sample with EPDS ≥ 10 between times 1 and 2 when comparing intervention (38.5% to 17.2%) vs control (31.9% to 29.4%), p = 0.008	None	N/A
		PPD awareness^2^ (structured questionnaire)	Participants reporting high PPD awareness (% intervention vs. control: 71.4% to 27%, p < 0.001		
		Perceived Social Support^2^ (structured questionnaire)	Participants reporting high perceived social support (% intervention vs control): 66.9% to 45.4%, p < 0.001		
Comrie-Thomson et al., 2022 [28]	12–15 post-intervention	Depressive symptom severity^1^ (EPDS mean)	RR = 0.66 (0.48 to 0.90), p = 0.008	None	N/A
		Prevalence of depressed mood^2^ (EPDS ≥ 12)	OR = 1.0 (0.6 to 1.4), p = 0.810		
		Health care seeking^2^	• Antenatal care attendance: OR 1.7 (1.1 to 2.6), p = 0.015 • Four or more antenatal care visits: OR 1.0 (0.4 to 2.4) p = 0.0938 • Timely postnatal care (mother): OR 15.7 (5.4 to 45.3), p < 0.0001 • Timely postnatal care (baby): OR 2.8 (1.7 to 4.8), p< 0.0001 • Couples HIV test during pregnancy: OR 2.1 (1.3 to • 3.4), p = 0.003 • Facility birth: OR 1.9 (0.6 to 5.9) p = 0.257		
		Women’s participation in decision making^2^ (Women’s Household Decision-making Power Scale)	• Major household purchases: OR 1.6 (0.9 to 2.9), p = 0.144 • Visits to family: OR 2.7 (1.8 to 4.2), p < 0.0001 • Health visits for woman: OR 1.6 (0.6 to 4.2), p = 0.356 • All three decisions: OR 2.0 (1.2 to 3.5), p = 0.012 • None of the decisions: OR 0.6 (0.3 to 1.3), p = 0.176		
		Couple relationship dynamics^2^ (total score Intimate Bond Measure)	MD -3.4 (-6.7 to 0.0), p = 0.048		
		Male practical support for their female coparent and baby^2^ (survey)	Support during pregnancy, OR • Obtain or prepare special foods for woman: 2.9 (1.1, 7.5) • Encourage woman to rest: 0.9 (0.2, 3.6) • Contribute to household chores: 4.2 (1.0, 18.1) • Accompany woman to ANC: 4.2 (1.7, 10.5) • Participate in ANC consultation, 2.1 (0.8, 5.0) Support around childbirth • Accompany woman to place of child- birth: 2.7 (1.5, 4.7) • Present during childbirth: 2.7 (1.1, 7.1) • Financial support for childbirth: 3.3 (0.3, 32.0) • Provide baby clothes or other items for childbirth: 1.0 (0.0, 21.9) Support for woman after birth† • Contribute to household chores: 2.4 (0.8, 6.9) • Encourage woman to breastfeed: 8.0 (2.0, 32.4) • Obtain or prepare special foods for woman: 9.2 (3.8, 22.1) Support for baby care • Bathe, dress, hold or play with baby: 68.8 (18.5, 256.1) • Hold or soothe baby at night: 1.9 (0.2, 14.5) • Take baby to health facility if sick: 9.1 (2.6, 31.4)		
		Men’s gender attitudes^2^ (mean score Gender Equitable Men scale)	MD -2.4 (-3.8 to -1.0), P = 0.005		
George et al., 2020 [33]	6–12 weeks postpartum	Prevalence of postpartum depression^1^ (CIS-R)	Intervention (14.06%) vs. control (22.62%), p = 0.30	None	None
		Depressive symptom severity^2^ (EPDS median)	No statistically significant difference, p = 0.31		
		Functioning^2^ (GAF < 70)	No statistically significant difference, p = 0.23		
Khan et al., 2017 [30]	2 months post-intervention	Depressive symptom severity^2^ (SRQ mean)	MD −1.08 (−2.75 to 0.59); (p = 0.20)	None	N/A
		Help-seeking for psychological distress^1^ (semi-structured interview)	Participants contacting LHWs for assistance (% intervention vs control): 71% vs 46%, *p* = .036		
		Social Support^2^ (Multidimensional Scale of Perceived Social Support)	MD 0.039 (−7.91, 7.98), p = 0.992		
Le Roux et al., 2020 [25]	6 months post-intervention	Depressive symptom severity^1^ (EPDS mean)	MD -0.9 (−1.7, −0.2), p < 0.05	Weight-for-age Z-score^2^ (WAZ)	MD: 0.1 (0.1, 0.3)
		Prevalence of depressed mood^2^ (EPDS>13)	OR 0.5 (0.3, 1.1)	Height-for-age Z-score^2^ (HAZ)	MD: -0.2 (-0.4, 0.1)
		Prevalence of probable clinical depression^2^ (EPDS ≥ 18)	OR 0.6 (0.2, 1.8)	Weight-for-height Z-score^2^ (WHZ)	MD: 0.3 (0.1, 0.6)
		Breastfeeding^2^	• Currently breastfeeding: OR 0.5 (0.1, 5.4) • Exclusively breast fed at 3 months: OR 1.6 (0.8, 3.3) • Exclusively breast fed at 6 months: OR 1.8 (1.1, 2.9)	Stunted^2^ (HAZ < -2SD)	OR 1.6 (0.6, 3.9)
		Mixing formula w/ baby porridge^2^	OR: 0.4 (0.2, 0.8)	Wasting^2^ (WHZ <-2SD)	OR 0.5 (0.3, 0.9)
		PMTCT^2^	• ARVs before/during pregnancy: 97.3% vs. 98.5% • Nevirapine at: 93.6% vs. 96.1% • ARVs post-birth: 93.1% vs. 96.1% • Child tested for HIV: 95.8% vs. 91.5% • Single Feeding Method: 63.2% vs. 68.8% • Child given Bactrim: 37.7% vs 17.1% (p < 0.01)	Underweight^2^ (WAZ <-2)	OR 0.4 (0.1, 1.0)
		Medical Visits^2^	• Hospital Visit: OR 0.5 (0.3, 1.1) • Clinic for Illness: OR 0.8 (0.6, 1.2) • Consulted with a traditional healer: OR: 0.2 (0.1, 0.8) • Consulted with a private doctor: OR 0.6 (0.3, 1.2) • Consulted with a private pharmacy: OR: 0.2 (0.1, 0.4)	Child illnesses^2^	• Diarrhea: OR 1.3 (0.9, 1.9) • Vomiting: OR 0.5 (0.3, 0.9) • Cough: OR 0.6 (0.4, 0.8) • SOB: OR 0.8 (0.5, 1.2) • Fever: OR 0.8 (0.6, 1.1) • Runny Nose: OR 0.7 (0.5, 0.9)
				Total Count of Symptoms during Past 2 Weeks^2^ (0–6)	IRR 0.8 (0.7, 0.9); p < 0.05
				Immunizations Up to Date^2^	OR 1.1 (0.8, 1.6)
				Vitamin A^2^	OR 1.3 (0.8, 2.0)
				Development^2^ (WHO Milestones)	On/Ahead Target vs. Behind: OR 1.3 (0.7, 2.6)
Maselko et al., 2020 [31]	36 months postpartum	Symptom severity^1^ (PHQ-9, total score)	SMD −0.13 (−0.33, 0.07)	Child socioemotional skills^1^ (SDQ-TD)	SMD −0·10 (−1·39, 1·19)
		Remission^1^ (PHQ-9 <10)	RR 1.00 (0.88, 1.13)	Development^2^ (BSITD III)	• Receptive Language: SMD 0.38 (-0.19, 0.96) • Fine Motor: SMD 0.03 (-0.83, 0.9)
		Prevalence of maternal major depressive episode^2^ (SCID)	RR 0.67 (0.43, 1.05)		
		Disability^2^ (WHO-DAS)	SMD −0.12 (−0.33, 0.09)		
Rahman et al. 2008 [32]	6–12 months postpartum	Symptom severity^2^ (HDRS)	• 6 months: MD -5.86 (-7.92, -3.80), p < 0.0001 • 12 months: MD -6.65 (-8.56, -4.74), p < 0.0001	Weight-for-age Z score^1^	• 6 months: MD -0.02 (-0.18, 0.14, p = 0.76 • 12 months: MD 0.07 (-0.08, 0.22, p = 0.37
		Prevalence of perinatal depression^2^ (SCID (DSM-IV)	• 6 months: OR 0.22 (0.14, 0.36), p < 0.0001 • 12 months: OR 0.23 (0.15, 0.36), p < 0.0001	Height-for-age Z-score^1^	• 6 months: MD 0.07 (-0.09, 0.23, p = 0.38 • 12 months: MD 0.017 (-0.02, 0.35, p = 0.07
		Disability^2^ (Brief Disability Questionnaire)	• 6 months: MD -1.80 (-2.38, -1.12), p < 0.0001 • 12 months: MD -2.88 (-3.66, -2.10), p< 0.0001	Diarrhea episodes at 12 months^2^	OR 0.6 (0.39, 0.98), p = 0.04
		Functioning^2^ (GAF)	• 6 months: MD 6.85 (4.73, 8.96), p < 0.0001 • 12 months: MD 8.27 (6.32, 10.31), p < 0.0001	Complete immunization at 12 months^2^	OR 2.5 (1.47, 4.72), p = 0.001
		Social support^2^ (MSPSS)	• 6 months: MD 6.71 (3.93, 9.48), p < 0.0001 • 12 months: MD 7.85 (5.43, 10.27), p < 0.0001		
		Exclusive Breastfeeding at 6 months ^2^	OR 1.6 (0.79, 3.18), p = 0.20		
		Contraceptive Use at 12 months^2^	OR 1.6 (1.20, 2.27), p = 0.002		
		Play Frequency^2^ w/ Infant at 12 months)	• Mother: OR 2.4 (2.07, 4.01), p < 0.0001 • Father: OR 1.9 (1.59, 4.15), p = 0.0001		
Sikander et al., 2019 [26]	3–6 months postpartum	Symptom severity^1^ (PHQ-9)	• 3 months: SMD –0.30 (–0.48, –0.11), p<0.0001 • 6 months: SMD –0.13 (–0.31, 0.06), p = 0.07	Weight-for-age Z score^2^	• 3 months: SMD -0.08 (-0.28, 0.11), p = 0.32 • 6 months: SMD -0.06 (-0.24, 0.13), p = 0.50
		Prevalence of remission^1^ (PHQ-9 <5)	• 3 months: PR 1.18 (1.06, 1.29), p = 0.001 • 6 months: PR 1.12 (0.95, 1.29); p = 0.14	Height-for-age Z score^2^	• 3 months: SMD 0.05 (-0.14, 0.24), p = 0.55 • 6 months: SMD 0.08 (-0.11, 0.26), p = 0.29
		Recovery from Depression (defined as PHQ-9 <5 at 3 and 6 months postpartum)	PR 1.36 (1.09, 1.63), p = 0.002		
		Disability^2^ (WHO-DAS)	• 3 months: SMD -0.15 (-0.34, 0.03), p < 0.0001 • 6 months: SMD -0.11 (-0.29, 0.08), p = 0.23		
		Number of days unable, work in last month^2^	• 3 months: SMD -0.07 (-0.26, 0.12), p = 0.35 • 6 months: SMD 0.02 (-0.17, 0.20), p = 0.71		
		Perceived Social Support^2^ (MSPSS)	• 3 months: SMD 0.10 (-0.08, 0.29), p = 0.12 • 6 months: SMD 0.20 (0.02, 0.39), p = 0.007		
		Exclusive breastfeeding^2^	• 3 months: PR 0.94 (0.77, 1.10), p = 0.48 • 6 months: PR 0.89 (0.38, 1.40), p = 0.69		
Stansert Katzen et al., 2020 [29]	0–24 months postpartum	Prevalence of probable depressive disorder^2^ (EPDS ≥13)	Trend for HV group mothers to show fewer depressive symptoms over time compared to SC; however, not statistically significant (estimate—0.55, SE—0.33; p < 0.98) • Birth (% control vs. intervention): 15 vs 17 • 3 months postpartum: 15 vs 15 • 6 months postpartum: 15 vs 12 • 9 months postpartum: 12 vs 11 • 12 months postpartum: 16 vs 9 • 24 months postpartum: 8 vs 6	Stunting	• Birth (% control vs. intervention): 7 vs 7 • 3 months postpartum: 3 vs 4 • 6 months postpartum: 7 vs 4 • 9 months postpartum: 7 vs 6 • 12 months postpartum: 7 vs 10 • 24 months postpartum: 7 vs 9
		Antenatal visits, completed four prior to delivery	OR 1.85 (1.14, 3.00), p = 0.012	Severe low-weight for age (WAZ < -2SD)	• Birth (% control vs intervention): 11 vs 7 • 3 months postpartum: 5 vs 3 • 6 months postpartum: 7 vs 2 • 9 months postpartum: 2 vs 5 • 12 months postpartum: 4 vs 4 • 24 months postpartum: 4 vs 2
		Exclusively breastfeeding at 3 months	OR 1.97 (1.20, 3.25), p = 0.008 • 3 months postpartum (% control vs. intervention): 17 vs 29 • 6 months postpartum: 18 vs. 25	Wasting (WHZ < -2SD)	• Birth (% control vs intervention): 19% vs 16% • 3 months postpartum: 10 vs 9 • 6 months postpartum: 5 vs 4 • 9 months postpartum: 4 vs 1 • 12 months postpartum: 0 vs 3 • 24 months postpartum: 3 vs 5
		PMTCT Tasks for Mothers living with HIV	• ART before birth (% control vs intervention): 3 vs 7 • NVP at birth: 91 vs 96 • PCR Testing, 3 months: 24 vs 36 • PCR Testing, 6 months: 51 vs 43	Mean WHO Developmental Score (SD)	• Birth (control vs intervention): - • 3 months postpartum: - • 6 months postpartum: 1.20 vs 1.36 • 9 months postpartum: 1.98 vs 1.97 • 12 months postpartum: 1.94 vs 1.96 • 24 months postpartum: 1.98 vs 2.0
		Traditional healer^2^, visited within 3 months	OR 2.33 (1.04, 5.20), p = 0.039)	Immunizations Up to Date^2^	• 3 months (% control vs intervention): 51 vs 45 • 6 months: 72 vs 75 • 9 months: 84 vs 83 • 12 months: 77 vs 66
				Child support grant	• Secured by 6 months (% control vs intervention): 63 vs 71 • Secured by 12 months: 76 vs 80
Stansert Katzen et al., 2021 [24]	12 months postpartum	Depressive symptom severity^1^ (EPDS mean)	MD -0.8 (-1.5, -0.1), p < 0.05	Weight-for-age Z-score (WAZ)^2^	MD: 0.1 (-0.1, 0.3)
		Prevalence of depressed mood^2^ (EPDS >13)	OR 0.8 (0.4, 1.6)	Height-for-age Z-score (HAZ)^2^	MD: -0.2 (-0.4, 0.02)
		Prevalence of probable clinical depression^2^ (EPDS ≥18)	OR 1.0 (0.3, 3.5)	Weight-for-height Z-score (WHZ)^2^	MD: -0.2 (-0.5, 0.1)
		Medical Visits^2^	• Hospital Visit: OR 0.9 (0.3, 3.1) • Clinic for Illness: OR 1.2 (0.8, 1.7) • Consulted with a traditional healer: OR: 0.5 (0.1, 1.5) • Consulted with a private doctor: OR 0.3 (0.1, 0.8) Consulted with a private pharmacy: OR: 0.4 (0.2, 0.6)	Stunted (HAZ < -2SD)^2^	OR 1.3 (0.6, 2.9)
				Wasting (WHZ <-2SD)^2^	OR 1.6 (0.4, 6.0)
				Malnourished (WAZ <-2)^2^	OR 0.5 (0.1, 1.5)
				Child illnesses^2^	• Diarrhea: OR 1.0 (0.7, 1.5) • Vomiting: OR 0.8 (0.4, 1.6) • Cough: OR 0.7 (0.5, 1.0) • SOB: OR 1.6 (0.8, 3.1) • Fever: OR 0.8 (0.5, 1.2) • Runny Nose: OR 0.7 (0.5, 1.1)
				Immunizations Up to Date^2^	OR 1.4 (0.7, 2.6)
				Development (WHO Milestones)^2^	On/Ahead Target vs. Behind: OR 2.1 (1.1, 4.1), p < 0.05
Tripathy et al., 2010 [34]	1–3 years	Depressive symptom severity^1^ (Kessler 10 item scale, measured 6 weeks after delivery)	• None–mild: OR 1.29 (0.68, 2.44) • Moderate: OR 0.74 (0.40, 1.37) • Severe: OR 1.29 (0.46, 3.64)	Neonatal mortality rate per 1000 livebirths^1^	OR 0·68 (0·59, 0·78), p < 0.0005
		Stillbirth rate per 1,000 births^2^	OR 1.05 (0.86, 1.28), p = 0.656		
		Perinatal mortality rate per 1,000 births^2^	OR 0.79 (0.69, 0.91), p < 0.0005		
		Maternal mortality ratio per 100,000 livebirths^2^	OR 0.70 (0.46, 1.07), p = 0.104		
		Births	• Any antenatal care: OR 1.60 (0.65, 3.92) ≥3 antenatal care visits: OR 0.69 (0.37, 1.26) • Iron tablets: OR 1.31 (0.62, 2.75) • Maternal tetanus-toxoid injection: OR 1.39 (0.85, 2.28) • Illness in pregnancy: OR 1.10 (0.71, 1.72) • Visited health facility in case of illness during pregnancy: OR 0.86 (0.46, 1.60) • Institutional Deliveries: OR 0.89 (0.51, 1.53) • Birth attended by formal provider (doctor or nurse): OR 0.81 (0.50, 1.31)		
		Home Deliveries	• Birth attended by traditional birth attendant: OR 0.84 (0.43, 1.64) • Birth attendant washed hands with soap: OR 2.07 (1.24, 3.45) • Safe-delivery kit use: OR 1.87 (1.11, 3.14) • Plastic sheet used: OR 3.74 (2.48, 5.65) • Cord tied with boiled thread: OR 3.02 (1.61, 5.65) • Cord cut with new or boiled blade: OR 1.35 (0.86, 2.12)		
		Livebirths (home deliveries)	• Cord undressed or dressed in antiseptic: OR 0.58 (0.27, 1.26) • Infant wiped within 30 min: OR 1.01 (0.43, 2.36) • Infant wrapped within 30 min: OR 0.78 (0.36, 1.66) • Infant not bathed in first 24 hours: OR 0.95 (0.44, 2.10)		
		Infants alive at 1 month	• Any of three infant illnesses (cough, fever, diarrhea): OR 0.67 (0.40, 1.12) • Care-seeking behavior in event of infant illness: OR 0.88 (0.97, 3.61) • Infant put, breast within 4h: OR 0.90 (0.38, 3.11) • vExclusive breastfeeding for first 6 weeks: OR 1.44 (0.89, 2.35)		

^1^Primary outcome

^2^Secondary outcome; OR–odds ratio; aMD–[adjusted] mean difference; aSMD–[adjusted] standardized mean difference; CI–confidence interval; SE–standard error; [I]–intervention; [C]–control; CHW–community health worker; CIS-R–Clinical Interview Schedule Revised; DAS–Disability Assessment Schedule; DSM-IV–Diagnostic and Statistical Manual IV; EPDS–Edinburgh Postnatal Depression Scale; GAF–Global Assessment of Functioning Scale; HAZ–Height-for-Age Z Score; HRDS–Hamilton Depression Rating Scale; MSPSS–Multidimensional Scale of Perceived Social Support; PHQ-9 –Patient Health Questionnaire; PMTCT–Prevention of mother-to-child transmission; PPD–postpartum depression; SCID–Structured Clinical Interview for DSM-IV; SRQ–Self-Reporting Questionnaire; WAZ—Weight-for-Age Z Score; WHO–World Health Organization; WHZ–Weight-for-Height Z Score

Measured outcomes were categorized as either maternal (e.g., disability, functioning, health-care behaviors, mother-infant interaction) or child outcomes (e.g., height, weight, cognitive development).

Eight of the nine studies included in this review identified maternal measures as primary outcomes. Six of the eight studies identified one or more depression-related outcomes as primary [24–28, 31, 33, 34], while the remaining two examined maternal help-seeking behaviors [30] and antenatal care visit attendance [29]. Only one study [32] examined child height and weight as the primary outcome.

While the outcome assessment tools and screening thresholds varied between studies, the depression-related outcomes were most frequently assessed using the EPDS [24, 25, 27–29, 33]. Other frequently used assessment tools included the WHO-Disability Assessment Score (DAS), the Global Assessment of Functioning Scale (GAF), and the WHO Motor Developmental Milestones.

### Control groups

Seven studies used standard antenatal care as their comparator group [24, 25, 27–30, 33, 34]. Two studies described the control group as receiving enhanced antenatal care [26, 31, 32]. In the study by Rahman and colleagues [32], enhanced antenatal care involved control participants receiving an equal number of home visits as the intervention group, although visits were by a CHW not trained in the intervention. Conversely, control participants in the study performed by Maselko, Sikander, and colleagues [26, 31] were provided standard antenatal care and informed of their depression screening results and how to seek the appropriate care.

### Effectiveness of interventions

#### Depression

Out of the nine studies examined in this review, five of them reported statistically significant improvements in depression-related outcomes among participants [24–28, 32]. Two interventions significantly reduced the prevalence of perinatal depression compared to usual care [27, 32], while the remaining three studies reported statistically significant reductions in symptom severity [24–26, 28].

#### Maternal wellbeing

Other indicators of maternal wellbeing yielded mixed results. Three studies [26, 27, 32] reported increased perceived social support among mothers receiving the intervention, while one study [37] found no difference between intervention and control groups. Two studies [26, 32], both employing the Thinking Healthy Program, indicated statistically significant improvements in maternal functioning and/or reduced maternal disability compared to enhanced antenatal care at 3–12 months postpartum. George and colleagues [33] found no statistically significant difference in maternal functioning between intervention and control arms.

### Other outcomes

#### Maternal health care utilization

Two studies [28, 29] revealed that mothers receiving the intervention were more likely to attend antenatal visits regularly compared to controls, while one study [34] showed no difference. Regarding prevention of mother-to-child HIV transmission (PMTCT) activities, both studies [25, 29] found no statistically significant difference in anti-retroviral adherence between intervention and control groups. However, Rahman and colleagues noted that mothers in the Thinking Healthy Program were more likely to use contraception actively at 12 months postpartum when compared to those receiving enhanced antenatal care [32].

#### Mother-Child interactions

Three studies [26, 32, 34] found no statistically significant difference in the proportion of women exclusively breastfeeding between intervention and control groups. In the context of childhood illness-related medical visits, two studies [25, 29] reported that intervention arm mothers were less likely to consult a traditional healer, while Tripathy and colleagues found no difference in care-seeking behavior [34]. Regarding childhood immunization, two studies [25, 29] found no difference between intervention and control groups, but le Roux and colleagues observed that mothers in the intervention arm were more likely to give their children HIV co-trimoxazole prophylaxis at 6 weeks postpartum when compared to women receiving standard antenatal care.

#### Parenting dynamics

Comrie et al. (2022) reported that intervention arm mothers were more involved in family decision-making, received increased male practical support, and experienced a less controlling relationship dynamic compared to the control arm. Rahman et al. (2008) found that infants born to mothers in the intervention group had more frequent interactions with both parents compared to control counterparts.

#### Child physical health and mortality

None of the studies found statistically significant differences in the height and weight of the children between intervention and control groups [24–26, 29, 32]. One study [25] reported a significantly lower likelihood of wasting in children born to mothers in the intervention arm when compared controls (OR 0.5, 95% CI 0.3 to 0.9). For symptoms of regular childhood illness (e.g., cough, diarrhea, vomiting), two studies [25, 32] revealed that infants in the intervention group had less frequent symptoms compared to the control group. One study [34] reported a statistically significant reduction (OR 0.68, 95% CI 0.59 to 0.78) in neonatal mortality in the intervention group compared to controls.

#### Child cognitive development

Mbereko and Men’s six-month outcomes showed no difference in achieving WHO Developmental Milestones [25], but 12-month outcomes indicated that children born to intervention group mothers were more likely to meet or surpass developmental targets [24].

### Intervention type and delivery

Overall, there are too few studies included in this review to draw reliable conclusions on the effectiveness of interventions to improve maternal wellbeing based on the type of intervention and delivery agent.

Nevertheless, a broad overview of the data included herein shows that psychoeducation and problem-solving therapy were the most used intervention elements and female CHWs were the most used agent of delivery. In addition, all three delivery methods–group, individual, both–demonstrated some evidence for their effectiveness in improving maternal wellbeing, although interventions that did not report improvements in depression-related outcomes were more likely to have been conducted via group-based sessions. Finally, more frequently delivered interventions, particularly those started during pregnancy and extending to 6–12 months postpartum, appear more likely to improve maternal wellbeing. Of the four studies that did not show improvements in symptom severity or remission for participants, one had two sessions in total [30], another lasted only three days [33], and a third had a relatively short (6 weeks) postpartum follow-up period [34]. Four out of five studies reporting improvement in depression-related outcomes began during pregnancy and extended into the postpartum period [24–27, 32].

Of note, loss to follow-up is particularly common in research conducted in rural communities, and many studies employed repeated house visits or telephone calls to minimize the number of participants who did not complete all intervention sessions, particularly for interventions of longer duration. Several studies also attempted to integrate interventions into the existing rural healthcare system by employing community health workers already well known in the community.

Risk of bias assessment (Tables 4 and 5)

**Table 4:** Risk of bias assessment using ROBINS-1 for Non-randomized studies.

Article	Domain 1: Confounding	Domain 2: Selection of Participants	Domain 3: Classification of Interventions	Domain 4: Deviations from Intended Intervention	Domain 5: Missing Data	Domain 6: Measurement of Outcomes	Domain 7: Selection of Reported Result	Overall Bias
Alfayumi et al. (2022)	Moderate	Moderate	Moderate	Low	Moderate	Moderate	Low	Moderate
George et al. (2020)	Serious	Low	Low	Low	NR	Low	Low	Serious
Stansert Katzen et al. (2020)	Moderate	Moderate	Low	Low	Moderate / NR	Moderate	Low	Moderate–Serious
Stansert Katzen et al. (2021)	Low	Low	Low	Low	Moderate	Moderate	Low	Low–Moderate
Le Roux et al. (2020)	Moderate	Low	Low	Low	Moderate	Moderate	Low	Moderate

NR–not reported

**Table 5:** Risk of bias assessment using RoB-2 for RCTs and Cluster-RCTs.

Article	Type of Study Design	Domain 1: Randomization Process	Domain 2: Deviations	Domain 3: Missing Outcome Data	Domain 4: Measurement of Outcome	Domain 5: Selection of Reported Result	Overall Bias
Khan et al. (2017)	RCT	Some concerns	Some concerns	Low	High	Low	High
Comrie-Thomson et al. (2022)	Cluster-RCT	Some concerns	Low	Low	Low	Low	Low
Rahman et al. (2008)	Cluster-RCT	Low	Low	Low	Low	Some concerns	Low
Sikander et al. (2019)	Cluster-RCT	Low	Low	Low	Low	Low	Low
Maselko et al. (2020)	Cluster-RCT	Some concerns	Low	High	Low	Low	Low
Tripathy et al. (2010)	Cluster-RCT	Some concerns	Low	Low	Some concerns	Low	Some concerns

Concerns about the randomization process were noted for four of the six assessed RCTs due to notable sociodemographic differences between intervention and control groups at baseline. Generally, concern for domain 2 (deviations) was low, except in one study where the same CHWs delivered both the intervention and control [30]. For domain 3, five RCTs provided adequate information and reported retention rates >85%, except one trial with a 63% retention rate at 36 months [31]. Measurement concerns were noted in two trials, one relying on self-reports from CHWs [30] and another with non-blinded outcome assessors [34]. Selective reporting concerns (domain 5) were low, except in one trial without a located protocol [32].

Non-randomized controlled trials in this review had a higher risk of bias and were determined to have at least a moderate risk of overall bias.

## Discussion

First and foremost, this review reveals limited evidence for the effectiveness of non-specialist delivered psychosocial interventions to significantly improve depression outcomes among women with perinatal depression living in rural communities. Only a few studies in this review reported any statistically significant differences in measured outcomes for this demographic, and the considerable overall risk of bias raises concerns about the validity and reproducibility of reported results. Furthermore, most studies did not focus exclusively on this participant population, and the lack of statistical power to analyze depression-related outcomes specifically in this demographic further reduced the body of evidence available for examination.

This overarching finding is consistent with the broader literature evaluating non-specialist interventions for common perinatal mental disorders conducted in urban and/or suburban areas. While some systematic reviews and meta-analyses on this topic have shown small, albeit promising results, the quality of evidence on which these findings are based is similarly poor. A Cochrane review reported that interventions delivered by lay health workers in LMICs might increase recovery in mothers suffering from perinatal depression but urged caution when considering this finding due to the preponderance of low-quality evidence [38]. Related studies have raised similar concerns [15, 39], and a systematic review of non-specialist delivered interventions for maternal mental health delivered in HICs noted that only half of the eligible trials presented information regarding treatment dosage and/or key supervision processes [14]. The relative lack of high-quality evidence reflected in this review and the broader literature more generally interferes with efforts to reliably improve perinatal mental health, particularly for those already suffering from significant symptoms.

Nevertheless, this review provides some evidence that interventions utilizing some combination of psychotherapeutic approaches, including problem-solving, behavioral activation, and cognitive restructuring, may be effective in treating women with perinatal depression living in rural communities. This finding is concordant with results of existing reviews and meta-analyses, which demonstrate psychotherapeutic approaches integrating interpersonal therapy or principles of CBT (e.g., cognitive restructuring, problem-solving) to be more effective in the prevention and treatment of perinatal depression than alternative approaches such as supportive counselling [14, 15, 40–42]. Similar findings are also reflected in the literature concerning adult major depressive disorder more generally; brief CBT and IPT-based psychotherapeutic approaches are more effective in the treatment of moderate to severe depression when compared to placebo [43] or usual care [44]. As such, future perinatal mental health interventions in rural communities should prioritize including such psychotherapeutic approaches in their design.

Secondly, this review indicates that non-specialist delivered psychosocial interventions do not significantly improve child outcomes, such as physical health and development, regardless of intervention type, delivery agent, or frequency. The association between perinatal depression and poor child health and development outcomes has been well established; nevertheless, few studies have been able to demonstrate that interventions for perinatal depression result in a significant improvement to child-related outcomes. While a recent meta-analysis found that non-specialist led interventions for common perinatal mental disorders were associated with better child cognitive development and improved growth indices [45], other studies have suggested otherwise. In fact, Cuijpers and colleagues found that psychological treatments for perinatal depression demonstrated no significant effects on the height and weight of offspring, results that mirror what is reported in this review [46]. As such, further exploration is needed to better understand what intervention components or approaches are necessary to reliably improve child outcomes, and future research teams should prioritize elucidating the mechanisms that underlie these complex interactions.

The notable exception to this finding, however, is the significant reduction in neonatal mortality, likely secondary to improved birth and hygiene practices, reported by Tripathy and colleagues. As such, it would be important to consider that peer-led group interventions of this nature may be of benefit in parts of the world where health literacy is low and early neonatal mortality rates are high due to unhygienic birth practices.

Third, this review also suggests that interventions delivered more frequently, over a more extended period, and initiated during pregnancy may be the most likely to significantly improve depression-related outcomes for women in rural communities. This finding must be interpreted cautiously as a review of the literature reveals little conclusive evidence as to the ideal dosage and timing of interventions for treating perinatal depression. Although a meta-analysis showed multiple-session interventions to be effective in preventing postnatal depression while those with a single session were not [47], other studies have found no association between treatment dosage and effect size [15] or have been unable to draw reliable conclusions due to poor reporting of key implementation details [14]. Previously published meta-analyses also found no association between intervention timing (e.g., during pregnancy vs postpartum) and effect sizes for depression-related outcomes [15, 48].

This review’s finding is somewhat at odds with the broader literature examining treatments for adult depression, an evaluation of which reveals strong supporting evidence for brief, low-intensity (LI) psychological interventions, particularly in the context of collaborative care and stepped-care models [49]. LI psychological interventions, abbreviated versions of evidenced-based therapies that can be delivered by workers with little to no formalized mental health training, are generally limited in terms of duration and frequency of contact (e.g., <6–10 distinct sessions) [50, 51]. Numerous systematic reviews and meta-analyses have demonstrated the effectiveness of LI interventions for treating a range of mental health conditions [38, 43, 52, 53], and their integration with stepped-care treatment models have been encouraged by the Lancet Commission for Global Mental Health and Sustainable Development as well as the WHO Mental Health Gap Action Plan [49, 54]. Stepped care models encourage the use of the least intensive, least expensive treatment first (e.g., non-specialist delivered LI psychological interventions) with the flexibility to provide more intensive care depending on individual patient needs or severity of presentation [55, 56]. As a result, this model of care ensures efficient allocation of resources and has been shown to effectively expand coverage of mental health care in resource-constrained settings such as LMICs [55, 57, 58]. While there are some concerns about the durability of LI treatment [59], stepped-care models integrating LI interventions appear to be a feasible and scalable way of providing effective depression treatment despite the limited duration and frequency of individualized sessions.

Despite the significant overlap in symptomatology between perinatal depression and depression in the general population, effective treatment for perinatal depression may differ from that of major depressive disorder due to the unique stressors and neurohormonal fluctuations characteristic of the postnatal period [60]. If, as this review suggests, longer and more intensive interventions are more effective in the treatment of perinatal depression for women living in rural communities, future implementation efforts must ensure that interventions remain both inexpensive and sustainable. This is particularly crucial in rural areas, which often have higher rates of poverty and significantly fewer mental health resources when compared to their urban or suburban counterparts [18, 61]. While this review is unable to draw conclusions as to the ideal format of delivery in rural communities due to the small number of studies included here, research suggests that both individual and group sessions are effective in the treatment of postpartum depression [15, 62]. Stepped-care models, like that discussed above, may also help ensure that women requiring more substantial, intensive care can access the care they require despite limited human resources and inadequate government funding. As such, adapting existing interventions to accommodate delivery in a group format and/or within a stepped-care system may provide an opportunity for more efficient and sustainable use of limited resources in rural communities.

In summary, this review allows us to make tentative conclusions based on a narrative synthesis of limited and low-quality data. While they provide important considerations relevant to the design and implementation of maternal mental health interventions for rural communities, the true value of this review lies in the considerable literature gap it reveals. While the evidence presented herein supports the effectiveness of the Thinking Healthy Program—a World Health Organization (WHO) endorsed intervention—in rural communities, there is a pressing need for additional research that specifically targets women with or screening positive for perinatal depression. As this review demonstrates, many studies evaluating non-specialist perinatal mental health interventions include participants with and without depression and thus fail to provide any reliable insight into the most effective intervention design for women already struggling with poor mental health.

This review’s strengths include independent screening, data extraction, and risk of bias assessment by a second reviewer, comprehensive efforts to ensure inclusiveness through expert consultation and citation searches, and strict adherence to the original protocol with minimal changes.

This review also has important limitations to consider, several of which are inherent to the individual studies and some to the methodological design of the review itself. First, this review was limited to papers available in English and did not search grey literature. Second, narrow inclusion criteria, particularly regarding the population and chosen time frame (<12 months postpartum) of the perinatal period, may have excluded studies, and some included studies were not specifically powered for the perinatal depression population. Note that the depression-related outcomes studies included in this review either 1) identified depression-related measures as secondary outcomes or 2) had broader participant inclusion criteria and thus only provided subgroup analyses for women scoring above a pre-specific threshold on self-report measures. As such, several studies included here were not powered to detect a difference between intervention and control groups within this population. There are no effect sizes or relevant p-values reported for these studies. Furthermore, few studies clearly reported the exact means of oversight employed to maintain fidelity of the intervention, information that significantly impacts researchers’ ability to accurately assess the effectiveness of each intervention and its replicability. And finally, the global heterogeneity in defining "rural" may lead to varied classifications in included studies. It is also possible that not all research conducted in rural areas is included here if study authors did not explicitly define setting.

Limitations of the studies included in this review included overall considerable risk of bias, especially in non-randomized trials (Table 3), and significant heterogeneity among studies prevented a meta-analysis due to variations in measurement tools, outcomes, follow-up duration, and intervention characteristics. Of note, control groups predominantly received standard perinatal care, which potentially exaggerated intervention effects in settings with limited healthcare access.

Despite these limitations, this review should be considered as a comprehensive overview of the relatively sparse literature examining interventions for perinatal depression in rural communities. Its rigorous methodological quality contrasts with the limited and low-quality data available, and recommendations to address this literature gap are discussed in further detail below.

### Implications of this review

Our review has implications for research, clinical practice, and policy. Women living in rural communities worldwide often lack access to basic mental health care [63], and more research is needed to better care for these underserved populations. Future maternal mental health research must prioritize interventions tailored for rural settings. High-quality randomized controlled trials should be conducted, emphasizing scalability, sustainability, and cost-effectiveness in these contexts. Future studies must prioritize and clearly report the steps taken to ensure fidelity of each intervention over time as this and consistent high-quality supervision is critical to effective non-specialist delivered mental health care [64].

In researching maternal mental health interventions within rural communities, it’s also crucial for researchers to acknowledge and address the many logistical barriers faced by rural women when accessing in-person care. This includes but is not limited to addressing issues such as long travel distances to health and mental health clinics, as well as the lack of infrastructure supporting these journeys [65, 66]. Understanding and addressing these logistical barriers are paramount in delivering effective mental health support within rural contexts.

Primary care providers (PCPs), especially in low-resource settings like rural areas, should be trained to recognize signs of common perinatal mental disorders (CPMDs). Routine screening for perinatal depression, facilitated by tools like EPDS or PHQ-9, is essential throughout the perinatal period, given that many women living in rural areas receive postpartum care through primary health services. Additionally, PCPs should be prepared to offer psychoeducation and referrals to specialist services for at-risk patients, as brief psychoeducation has shown modest positive effects on CPMD treatment and prevention [39, 47, 67]. Furthermore, PCPs must collaborate closely with their peripartum patients to devise practical and sustainable solutions to the myriad of logistical and financial barriers, several of which are referenced throughout this review, that women living in rural communities may encounter when seeking specialized mental health care.

Finally, policymakers should prioritize integrating maternal mental health into routine perinatal care to enhance access, coverage, and early detection [68–70]. Failure to do so is costly; untreated perinatal mental health conditions impose a $14 billion annual societal burden in the U.S. alone [71].

## Conclusion

Common perinatal mental disorders, including but not limited to perinatal depression, negatively impact mothers and their children across the world. This review presents some evidence to support the effectiveness of non-specialist delivered interventions to improve depression-related outcomes among women with or at risk for perinatal depression living in rural communities. Research suggests that women living in rural areas may be at higher risk for perinatal depression and anxiety than their urban counterparts [72], and the relative lack of high-quality studies included here demonstrates that further investment of resources to support rural maternal mental health research is urgently needed.

Finally, I’d like to thank the participants here took part in the research studies included here as without them this review wouldn’t have been possible.

## Supporting information

S1 Text (DOCX)
