# Supplementary material for: Non-specialist delivered psycho-social interventions for women with perinatal depression living in rural communities: A systematic review
Source: PLOS Glob Public Health. 2024 Jul 8;4(7):e0003031. doi: 10.1371/journal.pgph.0003031 (PMC11230560; doi:10.1371/journal.pgph.0003031)
Supplement: S1 Text — (DOCX) [file pgph.0003031.s001.docx]

**Supplementary Materials**

**Table A** PRISMA Checklist(s)

1a. PRISMA 2020 Abstracts Checklist

| **Section and Topic** | **Item #** | **Checklist item** | **Reported (Yes/No)** |
| --- | --- | --- | --- |
| **TITLE** | | |  |
| Title | 1 | Identify the report as a systematic review. | Y |
| **BACKGROUND** | | |  |
| Objectives | 2 | Provide an explicit statement of the main objective(s) or question(s) the review addresses. | Y |
| **METHODS** | | |  |
| Eligibility criteria | 3 | Specify the inclusion and exclusion criteria for the review. | Y |
| Information sources | 4 | Specify the information sources (e.g. databases, registers) used to identify studies and the date when each was last searched. | Y |
| Risk of bias | 5 | Specify the methods used to assess risk of bias in the included studies. | Y |
| Synthesis of results | 6 | Specify the methods used to present and synthesize results. | Y |
| **RESULTS** | | |  |
| Included studies | 7 | Give the total number of included studies and participants and summarize relevant characteristics of studies. | Y |
| Synthesis of results | 8 | Present results for main outcomes, preferably indicating the number of included studies and participants for each. If meta-analysis was done, report the summary estimate and confidence/credible interval. If comparing groups, indicate the direction of the effect (i.e. which group is favored). | Y |
| **DISCUSSION** | | |  |
| Limitations of evidence | 9 | Provide a summary of the limitations of the evidence included in the review (e.g. study risk of bias, inconsistency and imprecision). | Y |
| Interpretation | 10 | Provide a general interpretation of the results and important implications. | Y |
| **OTHER** | | |  |
| Funding | 11 | Specify the primary source of funding for the review. | Y |
| Registration | 12 | Provide the register name and registration number. | Y |

Page MJ, McKenzie JE, Bossuyt PM, Boutron I, Hoffmann TC, Mulrow CD, et al. The PRISMA 2020 statement: an updated guideline for reporting systematic reviews. BMJ 2021;372:n71. doi: 10.1136/bmj.n71

1b. PRISMA-S for Reporting Literature Searches in Systematic Reviews

| **Section and Topic** | **Item #** | **Checklist item** | **Reported (Y/N)** | **Location where item is reported** |
| --- | --- | --- | --- | --- |
| **TITLE** | | |  |  |
| Title | 1 | Identify the report as a systematic review. | Y | Title page |
| **ABSTRACT** | | |  |  |
| Abstract | 2 | See the PRISMA 2020 for Abstracts checklist. | Y | See previous page |
| **INTRODUCTION** | | |  |  |
| Rationale | 3 | Describe the rationale for the review in the context of existing knowledge. | Y | Page(s) 4 – 5 |
| Objectives | 4 | Provide an explicit statement of the objective(s) or question(s) the review addresses. | Y | Page(s) 5 – 6 |
| **METHODS** | | |  |  |
| Eligibility criteria | 5 | Specify the inclusion and exclusion criteria for the review and how studies were grouped for the syntheses. | Y | Page 7 |
| Information sources | 6 | Specify all databases, registers, websites, organizations, reference lists and other sources searched or consulted to identify studies. Specify the date when each source was last searched or consulted. | Y | Page 6, Supplementary Materials Table 4 |
| Search strategy | 7 | Present the full search strategies for all databases, registers, and websites, including any filters and limits used. | Y | Table 4 |
| Selection process | 8 | Specify the methods used to decide whether a study met the inclusion criteria of the review, including how many reviewers screened each record and each report retrieved, whether they worked independently, and if applicable, details of automation tools used in the process. | Y | Page 8 |
| Data collection process | 9 | Specify the methods used to collect data from reports, including how many reviewers collected data from each report, whether they worked independently, any processes for obtaining or confirming data from study investigators, and if applicable, details of automation tools used in the process. | Y | Page 9 |
| Data items | 10a | List and define all outcomes for which data were sought. Specify whether all results that were compatible with each outcome domain in each study were sought (e.g. for all measures, time points, analyses), and if not, the methods used to decide which results to collect. | Y | Protocol, Supplementary Materials Table 3 |
|  | 10b | List and define all other variables for which data were sought (e.g. participant and intervention characteristics, funding sources). Describe any assumptions made about any missing or unclear information. | Y | Protocol, Supplementary Materials Table 3 |
| Study risk of bias assessment | 11 | Specify the methods used to assess risk of bias in the included studies, including details of the tool(s) used, how many reviewers assessed each study and whether they worked independently, and if applicable, details of automation tools used in the process. | Y | Page 9, Table 3 |
| Effect measures | 12 | Specify for each outcome the effect measure(s) (e.g. risk ratio, mean difference) used in the synthesis or presentation of results. | Y | Table 3 |
| Synthesis methods | 13a | Describe the processes used to decide which studies were eligible for each synthesis (e.g. tabulating the study intervention characteristics and comparing against the planned groups for each synthesis (item #5)). | Y | Page(s) 8 – 9 |
|  | 13b | Describe any methods required to prepare the data for presentation or synthesis, such as handling of missing summary statistics, or data conversions. | N/A | N/A |
|  | 13c | Describe any methods used to tabulate or visually display results of individual studies and syntheses. | Y | Page 9 |
|  | 13d | Describe any methods used to synthesize results and provide a rationale for the choice(s). If meta-analysis was performed, describe the model(s), method(s) to identify the presence and extent of statistical heterogeneity, and software package(s) used. | Y | Page 9 |
|  | 13e | Describe any methods used to explore possible causes of heterogeneity among study results (e.g. subgroup analysis, meta-regression). | N/A | N/A |
|  | 13f | Describe any sensitivity analyses conducted to assess robustness of the synthesized results. | N/A | N/A |
| Reporting bias assessment | 14 | Describe any methods used to assess risk of bias due to missing results in a synthesis (arising from reporting biases). | N/A | N/A |
| Certainty assessment | 15 | Describe any methods used to assess certainty (or confidence) in the body of evidence for an outcome. | N/A | N/A |
| **RESULTS** | | |  |  |
| Study selection | 16a | Describe the results of the search and selection process, from the number of records identified in the search to the number of studies included in the review, ideally using a flow diagram. | Y | Page 10, Figure 1 |
|  | 16b | Cite studies that might appear to meet the inclusion criteria, but which were excluded, and explain why they were excluded. | Reasons for exclusion | Figure 1 |
| Study characteristics | 17 | Cite each included study and present its characteristics. | Y | Page(s) 10 – 11 |
| Risk of bias in studies | 18 | Present assessments of risk of bias for each included study. | Y | Table 3 |
| Results of individual studies | 19 | For all outcomes, present, for each study: (a) summary statistics for each group (where appropriate) and (b) an effect estimate and its precision (e.g. confidence/credible interval), ideally using structured tables or plots. | Y | Table 1 |
| Results of syntheses | 20a | For each synthesis, briefly summarize the characteristics and risk of bias (RoB) among contributing studies. | Y | Page(s) 10 – 11, Table 3 |
|  | 20b | Present results of all statistical syntheses conducted. If meta-analysis was done, present for each the summary estimate and its precision (e.g. confidence/credible interval) and measures of statistical heterogeneity. If comparing groups, describe the direction of the effect. | N/A | N/A |
|  | 20c | Present results of all investigations of possible causes of heterogeneity among study results. | N/A | N/A |
|  | 20d | Present results of all sensitivity analyses conducted to assess the robustness of the synthesized results. | N/A | N/A |
| Reporting biases | 21 | Present assessments of risk of bias due to missing results (arising from reporting biases) for each synthesis assessed. | Y | Table 3 |
| Certainty of evidence | 22 | Present assessments of certainty (or confidence) in the body of evidence for each outcome assessed. | N |  |
| **DISCUSSION** | | |  |  |
| Discussion | 23a | Provide a general interpretation of the results in the context of other evidence. | Y | Page(s) 14 – 16 |
|  | 23b | Discuss any limitations of the evidence included in the review. | Y | Page(s) 17 – 18 |
|  | 23c | Discuss any limitations of the review processes used. | Y | Page(s) 17 – 18 |
|  | 23d | Discuss implications of the results for practice, policy, and future research. | Y | Page(s) 18 – 19 |
| **OTHER INFORMATION** | | |  |  |
| Registration and protocol | 24a | Provide registration information for the review, including register name and registration number, or state that the review was not registered. | Y | Page 6 |
|  | 24b | Indicate where the review protocol can be accessed, or state that a protocol was not prepared. | Y | Page 6 |
|  | 24c | Describe and explain any amendments to information provided at registration or in the protocol. | Y | Page 6 |
| Support | 25 | Describe sources of financial or non-financial support for the review, and the role of the funders or sponsors in the review. | Y | Title Page |
| Competing interests | 26 | Declare any competing interests of review authors. | N/A | N/A |
| Availability of data, code, and other materials | 27 | Report which of the following are publicly available and where they can be found: template data collection forms; data extracted from included studies; data used for all analyses; analytic code; any other materials used in the review. | Y | Supplementary Materials Table 3 |

Page MJ, McKenzie JE, Bossuyt PM, Boutron I, Hoffmann TC, Mulrow CD, et al. The PRISMA 2020 statement: an updated guideline for reporting systematic reviews. BMJ 2021;372:n71. doi: 10.1136/bmj.n71

**Table B** Screening and Selection Tool

Title: (72)

Author:

Journal (Year Published):

|  | INCLUDE | EXCLUDE |
| --- | --- | --- |
| Population^[[1]](#footnote-1)^ | Women who are either (a) **currently pregnant** or (b) **within 12 months** of delivery at time of intervention entry  Participants meet criteria for perinatal depression (PD) (as determined by individual study) using either (a) **standardized measures** (e.g., EPDS)^[[2]](#footnote-2)^ or (b) **diagnostic interview** | Women outside of the perinatal period^[[3]](#footnote-3)^  PD identified via other means (i.e., did not use standardized measure or diagnostic interview)  Participants with severe medical diagnoses^[[4]](#footnote-4)^ or severe developmental conditions (e.g., cerebral palsy) |
| Interventions^[[5]](#footnote-5)^ | Primary agent of delivery = non-specialist^[[6]](#footnote-6)^  Intervention evaluated (a) depressive symptomatology or (b) depression remission as primary or secondary outcome^[[7]](#footnote-7)^ | Primary agent of delivery = mental health specialist^[[8]](#footnote-8)^  Interventions that evaluated pharmacologic interventions *only*  Interventions conducted purely online  Self-help interventions w/o an active provider component  Interventions w/o a mental health component^[[9]](#footnote-9)^ |
| Comparators | Treatment as usual, no care, placebo, or any other intervention (including pharmacologic) | No comparison group |
| Outcomes | Include if one or more was evaluated as either a primary or secondary measure via diagnostic interviews or validated self-report symptom tools:  Depressive symptomatology  Depression remission | Studies that do not report any outcome specified in the inclusion criteria |
| Study Design | RCT OR quasi-experimental (e.g., cohort, case-control, etc.)  Peer-reviewed, published literature | Any other study designs  Not peer-reviewed and/or unpublished literature |
| Setting^[[10]](#footnote-10)^ | Studies in which authors reported the setting of their intervention to be rural  OR  Studies in which authors reported the participants to be from a rural/remote communities/population | Studies conducted in urban, peri-urban, or suburban settings  Author fails to specify location of intervention |
| Other | Studies published in English |  |

Possible Reason(s) for Exclusion:

- Ineligible setting (not conducted in rural location or with rural participants)
- Ineligible population (women outside perinatal window)
- Ineligible outcome(s)
- Ineligible publication type (e.g., peer-reviewed and/or unpublished)
- Ineligible study design (i.e., not RCT or quasi-experimental study design)
- Ineligible primary agent of delivery (e.g., mental health specialist)
- No comparator
- Unable to obtain text after repeated attempts
- Abstract only
- No intervention trailed
- Non-English

Notes:

**Table C** Data Extraction Categories

| Study Details | Author |
| --- | --- |
|  | Year of Publication |
|  | DOI |
|  | Study Aim(s) |
|  | Country |
|  | Study Setting |
|  | Study Design |
|  | Unit of Randomization |
| Participants | Total Number of Participants |
|  | Type of Participants |
|  | Number of Intervention Participants, n = |
|  | Number of Control Participants, n = |
|  | Inclusion Criteria |
|  | Exclusion Criteria |
|  | Mean Age (SD), Age Range |
|  | Demographic Differences between Int and Control |
|  | PD Identification Measure (e.g., EPDS) |
|  | Cut-off Score for Screening Tools |
|  | Physical and Mental Comorbidities |
| Intervention | Type |
|  | Timing |
|  | Content |
|  | Physical or Information Materials |
|  | Agent of Delivery |
|  | Training for Agent of Delivery |
|  | Comparator |
|  | Intervention Target |
|  | Delivery Format (e.g., in-person) |
|  | Group vs. Individual Format |
|  | Number and Duration of Sessions |
|  | Frequency of Sessions |
| Results | Primary Outcomes |
|  | Secondary Outcomes |
|  | Time to Follow-up |
|  | Subgroup Analyses |
|  | Main Findings |
|  | Effect Sizes |
| Additional Notes | (e.g., conflict of interest, funding sources, etc.) |

For Excel document of data extraction sheet, please contact corresponding author.

**Table D** Search Strategies

| **Date of search**: | **Interface:** | **Database:** | **Database dates:** | **Initial Count:** | **Limits/filters used?** |
| --- | --- | --- | --- | --- | --- |
| 1/23/23 | OVID | Embase | 1974 to 2023 Jan 20 | 581 | None |
| 1/23/23 | OVID | Medline | 1946 to Jan 20, 2023 | 473 | None |
| 1/23/23 | OVID | PsycINFO | 1806 to Jan Week 2 2023 | 285 | None |
| 1/23/23 | OVID | Global Health | 1910 to 2023 Week 03 | 491 | None |
| 1/23/23 | EBSCO | CINAHL | 1937 to Jan 22 2023 | 233 | None |
| 1/23/23 | Cochrane | CENTRAL | Issue 1 of 12, Jan 2023 | 141 | None |
| 1/24/23 | N/A | Global Index Medicus | 1972 – present | 489 | English |
| **Total(s):** | Total uploaded to Rayyan: 2,693  Number of duplicates removed by Rayyan: 1,043 | | | | |

Ovid Embase <1974 to 2023 January 20>

| 1 | exp perinatal depression/ | 8051 |
| --- | --- | --- |
| 2 | exp puerperal depression/ | 6399 |
| 3 | ((matern* or prenatal* or pre-natal* or antenatal* or ante-natal* or antepartum* or ante-partum* or perinatal* or peri-natal* or pregnan* or postpartum or post-partum or peripartum or peri-partum or postnatal* or post-natal* or puerper*) adj3 (depress* or dysthymi* or MDD or distress or 'affective symptom*' or 'baby blues' or 'disorder*' or CMD or 'low mood*')).mp. | 58917 |
| 4 | 1 or 2 or 3 | 58917 |
| 5 | exp rural population/ | 54419 |
| 6 | rural health care/ | 14406 |
| 7 | rural health/ | 1880 |
| 8 | rural area/ | 66130 |
| 9 | medically underserved/ | 1587 |
| 10 | rural hospitals/ | 1097 |
| 11 | (rural* or remot* or nonmetropolitan or non-metropolitan or suburb*).mp. | 368574 |
| 12 | (rural adj (health* or medicine or medical care or medical service?)).mp. | 21830 |
| 13 | ((rural or remote or nonmetropolitan or non metropolitan or suburb* or developing or less* developed or under developed or underdeveloped or middle income or low* income or underserved or under served or deprived or poor) adj3 (communit* or area? or village* or town? or count* or region? or province? or setting*)).mp. | 388150 |
| 14 | ((rural or remote or nonmetropolitan or non metropolitan or suburb* or middle income or low* income or underserved or under served or deprived or poor or village* or town) adj3 population*).mp. | 83614 |
| 15 | ((rural or remote or nonmetropolitan or non metropolitan or suburb* or village* or town) adj3 (clinic? or hospital? or facility or facilities or health* center? or health* centre? or health care center? or health care centre? or medical center? or medical centre?)).mp. | 22991 |
| 16 | ((shortage? or understaffed or under staffed) adj3 area?).mp. | 915 |
| 17 | 5 or 6 or 7 or 8 or 9 or 10 or 11 or 12 or 13 or 14 or 15 or 16 | 608690 |
| 18 | Allied Health Personnel/ | 14809 |
| 19 | Community Health Aides/ | 9025 |
| 20 | Nurses Aides/ | 4716 |
| 21 | Psychiatric Aides/ | 14809 |
| 22 | Caregivers/ | 84626 |
| 23 | Voluntary Workers/ | 5099 |
| 24 | Community Networks/ | 54665 |
| 25 | exp Self-Help Groups/ | 14524 |
| 26 | Social Support/ | 109180 |
| 27 | Health Manpower/ | 3114 |
| 28 | "Personnel Staffing and Scheduling"/ | 55797 |
| 29 | (lay adj3 (worker? or visitor? or attendant? or aid* or support* or person* or helper? or carer? or caregiver? or care giver? or counselor? or counsellor? or assistant? or staff)).mp. | 3398 |
| 30 | ((voluntary or volunteer?) adj3 (worker? or visitor? or attendant? or aid* or support* or person* or helper? or carer? or caregiver? or care giver? or consultant? or advisor? or counselor? or counsellor? or assistant? or staff)).mp. | 10770 |
| 31 | (untrained adj3 (worker? or visitor? or attendant? or aid* or support* or person* or helper? or carer? or caregiver? or care giver? or consultant? or advisor? or counselor? or counsellor? or assistant? or staff or nurse? or doctor? or physician? or therapist?)).mp. | 1027 |
| 32 | (trained adj3 (worker? or visitor? or attendant? or aid* or support* or person* or helper? or carer? or caregiver? or care giver? or consultant? or advisor? or counselor? or counsellor? or assistant? or staff or nurse? or doctor? or physician? or therapist?)).mp. | 36755 |
| 33 | (unlicensed adj3 (worker? or visitor? or attendant? or aid* or support* or person* or helper? or carer? or caregiver? or care giver? or consultant? or advisor? or counselor? or counsellor? or assistant? or staff or nurse? or doctor? or physician? or therapist?)).mp. | 476 |
| 34 | ((nonprofessional? or non professional?) adj3 (worker? or visitor? or attendant? or aid* or support* or person* or helper? or carer? or caregiver? or care giver? or consultant? or advisor? or counselor? or counsellor? or assistant? or staff)).mp. | 637 |
| 35 | ((non medical or non health or non healthcare or non health care) adj3 (worker? or visitor? or attendant? or aid* or support* or person* or helper? or carer? or caregiver? or care giver? or consultant? or advisor? or counselor? or counsellor? or assistant? or staff)).mp. | 1789 |
| 36 | (community adj3 (worker? or visitor? or attendant? or aid* or support* or person* or helper? or carer? or caregiver? or care giver? or consultant? or advisor? or counselor? or counsellor? or assistant? or staff)).mp. | 46347 |
| 37 | (paraprofessional? or paramedic or paramedics or paramedical worker? or paramedical personnel or allied health personnel or allied health worker? or support worker? or non specialist? or specially trained or barefoot doctor? or nurs* aid* or psychiatric aid* or psychiatric attendant? or social worker? or teacher? or school staff or trainer?).mp. | 153318 |
| 38 | ((health* or medical*) adj3 (auxiliary or auxiliaries)).mp. | 9506 |
| 39 | (nurs* adj1 (auxiliary or auxiliaries)).mp. | 744 |
| 40 | (informal adj (caregiver? or care giver? or carer?)).mp. | 6768 |
| 41 | (self help group? or support group?).mp. | 23788 |
| 42 | ((social or psychosocial) adj (care or support)).mp. | 163137 |
| 43 | (village adj3 worker?).mp. | 702 |
| 44 | community based.mp. | 95589 |
| 45 | (community adj3 intervention?).mp. | 15722 |
| 46 | community network?.mp. | 1141 |
| 47 | ((health or health care or healthcare) adj manpower).mp. | 1085 |
| 48 | human resources.mp. | 15118 |
| 49 | (task? adj3 shift*).mp. | 3835 |
| 50 | (task? adj3 shar*).mp. | 1996 |
| 51 | (staff* adj3 chang*).mp. | 3095 |
| 52 | 18 or 19 or 20 or 21 or 22 or 23 or 24 or 25 or 26 or 27 or 28 or 29 or 30 or 31 or 32 or 33 or 34 or 35 or 36 or 37 or 38 or 39 or 40 or 41 or 42 or 43 or 44 or 45 or 46 or 47 or 48 or 49 or 50 or 51 | 698943 |
| 53 | 4 and 17 and 52 | 581 |

Ovid MEDLINE(R) ALL <1946 to January 20, 2023>

| 1 | exp depression, postpartum/ | 7183 |
| --- | --- | --- |
| 2 | ((matern* or prenatal* or pre-natal* or antenatal* or ante-natal* or antepartum* or ante-partum* or perinatal* or peri-natal* or pregnan* or postpartum or post-partum or peripartum or peri-partum or postnatal* or post-natal* or puerper*) adj3 (depress* or dysthymi* or MDD or distress or 'affective symptom*' or 'baby blues' or 'disorder*' or CMD or 'low mood*')).mp. | 43246 |
| 3 | 1 or 2 | 43246 |
| 4 | exp rural population/ | 68486 |
| 5 | exp rural health services/ | 13962 |
| 6 | exp rural health/ | 23879 |
| 7 | hospitals, rural/ | 5202 |
| 8 | (rural* or remot* or nonmetropolitan or non-metropolitan or suburb*).mp. | 309984 |
| 9 | (rural adj (health* or medicine or medical care or medical service?)).mp. | 41642 |
| 10 | ((rural or remote or nonmetropolitan or non metropolitan or suburb* or developing or less* developed or under developed or underdeveloped or middle income or low* income or underserved or under served or deprived or poor) adj3 (communit* or area? or village* or town? or count* or region? or province? or setting*)).mp. | 303487 |
| 11 | ((rural or remote or nonmetropolitan or non metropolitan or suburb* or middle income or low* income or underserved or under served or deprived or poor or village* or town) adj3 population*).mp. | 92852 |
| 12 | ((rural or remote or nonmetropolitan or non metropolitan or suburb* or village* or town) adj3 (clinic? or hospital? or facility or facilities or health* center? or health* centre? or health care center? or health care centre? or medical center? or medical centre?)).mp. | 19219 |
| 13 | ((shortage? or understaffed or under staffed) adj3 area?).mp. | 763 |
| 14 | 4 or 5 or 6 or 7 or 8 or 9 or 10 or 11 or 12 or 13 | 508669 |
| 15 | Allied Health Personnel/ | 12889 |
| 16 | exp Community Health Aides/ | 6461 |
| 17 | Nurses Aides/ | 4387 |
| 18 | Psychiatric Aides/ | 414 |
| 19 | Caregivers/ | 48375 |
| 20 | Voluntary Workers/ | 10737 |
| 21 | Community Networks/ | 7178 |
| 22 | exp Self-Help Groups/ | 10673 |
| 23 | Social Support/ | 77990 |
| 24 | Health Manpower/ | 14338 |
| 25 | "Personnel Staffing and Scheduling"/ | 17963 |
| 26 | (lay adj3 (worker? or visitor? or attendant? or aid* or support* or person* or helper? or carer? or caregiver? or care giver? or counselor? or counsellor? or assistant? or staff)).mp. | 2658 |
| 27 | ((voluntary or volunteer?) adj3 (worker? or visitor? or attendant? or aid* or support* or person* or helper? or carer? or caregiver? or care giver? or consultant? or advisor? or counselor? or counsellor? or assistant? or staff)).mp. | 4579 |
| 28 | (untrained adj3 (worker? or visitor? or attendant? or aid* or support* or person* or helper? or carer? or caregiver? or care giver? or consultant? or advisor? or counselor? or counsellor? or assistant? or staff or nurse? or doctor? or physician? or therapist?)).mp. | 859 |
| 29 | (trained adj3 (worker? or visitor? or attendant? or aid* or support* or person* or helper? or carer? or caregiver? or care giver? or consultant? or advisor? or counselor? or counsellor? or assistant? or staff or nurse? or doctor? or physician? or therapist?)).mp. | 24002 |
| 30 | (unlicensed adj3 (worker? or visitor? or attendant? or aid* or support* or person* or helper? or carer? or caregiver? or care giver? or consultant? or advisor? or counselor? or counsellor? or assistant? or staff or nurse? or doctor? or physician? or therapist?)).mp. | 438 |
| 31 | ((nonprofessional? or non professional?) adj3 (worker? or visitor? or attendant? or aid* or support* or person* or helper? or carer? or caregiver? or care giver? or consultant? or advisor? or counselor? or counsellor? or assistant? or staff)).mp. | 550 |
| 32 | ((non medical or non health or non healthcare or non health care) adj3 (worker? or visitor? or attendant? or aid* or support* or person* or helper? or carer? or caregiver? or care giver? or consultant? or advisor? or counselor? or counsellor? or assistant? or staff)).mp. | 1206 |
| 33 | (community adj3 (worker? or visitor? or attendant? or aid* or support* or person* or helper? or carer? or caregiver? or care giver? or consultant? or advisor? or counselor? or counsellor? or assistant? or staff)).mp. | 31829 |
| 34 | (paraprofessional? or paramedic or paramedics or paramedical worker? or paramedical personnel or allied health personnel or allied health worker? or support worker? or non specialist? or specially trained or barefoot doctor? or nurs* aid* or psychiatric aid* or psychiatric attendant? or social worker? or teacher? or school staff or trainer?).mp. | 105957 |
| 35 | ((health* or medical*) adj3 (auxiliary or auxiliaries)).mp. | 575 |
| 36 | (nurs* adj1 (auxiliary or auxiliaries)).mp. | 720 |
| 37 | (informal adj (caregiver? or care giver? or carer?)).mp. | 5425 |
| 38 | (self help group? or support group?).mp. | 16941 |
| 39 | ((social or psychosocial) adj (care or support)).mp. | 117937 |
| 40 | (village adj3 worker?).mp. | 649 |
| 41 | community based.mp. | 77544 |
| 42 | (community adj3 intervention?).mp. | 12327 |
| 43 | community network?.mp. | 7921 |
| 44 | ((health or health care or healthcare) adj manpower).mp. | 885 |
| 45 | human resources.mp. | 12761 |
| 46 | (task? adj3 shift*).mp. | 3102 |
| 47 | (task? adj3 shar*).mp. | 1886 |
| 48 | (staff* adj3 chang*).mp. | 2030 |
| 49 | 15 or 16 or 17 or 18 or 19 or 20 or 21 or 22 or 23 or 24 or 25 or 26 or 27 or 28 or 29 or 30 or 31 or 32 or 33 or 34 or 35 or 36 or 37 or 38 or 39 or 40 or 41 or 42 or 43 or 44 or 45 or 46 or 47 or 48 | 459212 |
| 50 | 3 and 14 and 49 | 473 |

Ovid_APA PsycInfo <1806 to January Week 2 2023>

| 1 | exp postpartum depression/ | 5667 |
| --- | --- | --- |
| 2 | ((matern* or prenatal* or pre-natal* or antenatal* or ante-natal* or antepartum* or ante-partum* or perinatal* or peri-natal* or pregnan* or postpartum or post-partum or peripartum or peri-partum or postnatal* or post-natal* or puerper*) adj3 (depress* or dysthymi* or MDD or distress or 'affective symptom*' or 'baby blues' or 'disorder*' or CMD or 'low mood*')).mp. | 18802 |
| 3 | 1 or 2 | 18802 |
| 4 | exp rural environments/ | 21271 |
| 5 | exp rural health/ | 943 |
| 6 | (rural* or remot* or nonmetropolitan or non-metropolitan or suburb*).mp. | 78285 |
| 7 | (rural adj (health* or medicine or medical care or medical service?)).mp. | 4508 |
| 8 | ((rural or remote or nonmetropolitan or non metropolitan or suburb* or developing or less* developed or under developed or underdeveloped or middle income or low* income or underserved or under served or deprived or poor) adj3 (communit* or area? or village* or town? or count* or region? or province? or setting*)).mp. | 58135 |
| 9 | ((rural or remote or nonmetropolitan or non metropolitan or suburb* or middle income or low* income or underserved or under served or deprived or poor or village* or town) adj3 population*).mp. | 14851 |
| 10 | ((rural or remote or nonmetropolitan or non metropolitan or suburb* or village* or town) adj3 (clinic? or hospital? or facility or facilities or health* center? or health* centre? or health care center? or health care centre? or medical center? or medical centre?)).mp. | 2437 |
| 11 | ((shortage? or understaffed or under staffed) adj3 area?).mp. | 202 |
| 12 | 4 or 5 or 6 or 7 or 8 or 9 or 10 or 11 | 110932 |
| 13 | Allied Health Personnel/ | 1288 |
| 14 | Paraprofessional personnel/ | 1611 |
| 15 | Nonprofessional personnel/ | 177 |
| 16 | Psychiatric Aides/ | 179 |
| 17 | Caregivers/ | 34744 |
| 18 | Volunteers/ | 5810 |
| 19 | Home Care Personnel/ | 527 |
| 20 | Support Groups/ | 4549 |
| 21 | Social Support/ | 42049 |
| 22 | (lay adj3 (worker? or visitor? or attendant? or aid* or support* or person* or helper? or carer? or caregiver? or care giver? or counselor? or counsellor? or assistant? or staff)).mp. | 2000 |
| 23 | ((voluntary or volunteer?) adj3 (worker? or visitor? or attendant? or aid* or support* or person* or helper? or carer? or caregiver? or care giver? or consultant? or advisor? or counselor? or counsellor? or assistant? or staff)).mp. | 3157 |
| 24 | (untrained adj3 (worker? or visitor? or attendant? or aid* or support* or person* or helper? or carer? or caregiver? or care giver? or consultant? or advisor? or counselor? or counsellor? or assistant? or staff or nurse? or doctor? or physician? or therapist?)).mp. | 311 |
| 25 | (trained adj3 (worker? or visitor? or attendant? or aid* or support* or person* or helper? or carer? or caregiver? or care giver? or consultant? or advisor? or counselor? or counsellor? or assistant? or staff or nurse? or doctor? or physician? or therapist?)).mp. | 7493 |
| 26 | (unlicensed adj3 (worker? or visitor? or attendant? or aid* or support* or person* or helper? or carer? or caregiver? or care giver? or consultant? or advisor? or counselor? or counsellor? or assistant? or staff or nurse? or doctor? or physician? or therapist?)).mp. | 109 |
| 27 | ((nonprofessional? or non professional?) adj3 (worker? or visitor? or attendant? or aid* or support* or person* or helper? or carer? or caregiver? or care giver? or consultant? or advisor? or counselor? or counsellor? or assistant? or staff)).mp. | 736 |
| 28 | ((non medical or non health or non healthcare or non health care) adj3 (worker? or visitor? or attendant? or aid* or support* or person* or helper? or carer? or caregiver? or care giver? or consultant? or advisor? or counselor? or counsellor? or assistant? or staff)).mp. | 252 |
| 29 | (community adj3 (worker? or visitor? or attendant? or aid* or support* or person* or helper? or carer? or caregiver? or care giver? or consultant? or advisor? or counselor? or counsellor? or assistant? or staff)).mp. | 20204 |
| 30 | (paraprofessional? or paramedic or paramedics or paramedical worker? or paramedical personnel or allied health personnel or allied health worker? or support worker? or non specialist? or specially trained or barefoot doctor? or nurs* aid* or psychiatric aid* or psychiatric attendant? or social worker? or teacher? or school staff or trainer?).mp. | 278609 |
| 31 | ((health* or medical*) adj3 (auxiliary or auxiliaries)).mp. | 53 |
| 32 | (nurs* adj1 (auxiliary or auxiliaries)).mp. | 131 |
| 33 | (informal adj (caregiver? or care giver? or carer?)).mp. | 3087 |
| 34 | (self help group? or support group?).mp. | 13751 |
| 35 | ((social or psychosocial) adj (care or support)).mp. | 99655 |
| 36 | (village adj3 worker?).mp. | 79 |
| 37 | community based.mp. | 35520 |
| 38 | (community adj3 intervention?).mp. | 7503 |
| 39 | community network?.mp. | 1847 |
| 40 | ((health or health care or healthcare) adj manpower).mp. | 500 |
| 41 | human resources.mp. | 6484 |
| 42 | (task? adj3 shift*).mp. | 2338 |
| 43 | (task? adj3 shar*).mp. | 1540 |
| 44 | (staff* adj3 chang*).mp. | 1137 |
| 45 | 13 or 14 or 15 or 16 or 17 or 18 or 19 or 20 or 21 or 22 or 23 or 24 or 25 or 26 or 27 or 28 or 29 or 30 or 31 or 32 or 33 or 34 or 35 or 36 or 37 or 38 or 39 or 40 or 41 or 42 or 43 or 44 | 481344 |
| 46 | 3 and 12 and 45 | 285 |

Global Health <1910 to 2023 Week 03>

| 1 | ((matern$ or prenatal$ or pre-natal$ or antenatal$ or ante-natal$ or antepartum$ or ante-partum$ or perinatal$ or peri-natal$ or pregnan$ or postpartum$ or post-partum$ or peripartum$ or peri-partum$ or postnatal$ or post-natal$ or puerper$) adj3 (depress$ or dysthymi$ or MDD or distress or 'affective symptom$' or 'baby blues' or disorder$ or CMD or 'low mood$')).mp. | 6823 |
| --- | --- | --- |
| 2 | exp rural population/ | 1542 |
| 3 | exp rural areas/ | 84068 |
| 4 | rural health/ | 7321 |
| 5 | rural welfare/ | 65 |
| 6 | (rural$ or remot$ or nonmetropolitan or non-metropolitan or suburb$).mp. | 147197 |
| 7 | (rural adj (health$ or medicine or medical care or medical service$)).mp. | 37239 |
| 8 | ((rural$ or remot$ or nonmetropolitan or non metropolitan or suburb$ or developing or less$ developed or under developed or underdeveloped or middle income or low$ income or underserved or under served or deprived or poor) adj3 (communit$ or area$ or village$ or town$ or count$ or region$ or province$ or setting$)).mp. | 1242293 |
| 9 | ((rural$ or remot$ or nonmetropolitan or non metropolitan or suburb$ or middle income or low$ income or underserved or under served or deprived or poor or village$ or town) adj3 population$).mp. | 17226 |
| 10 | ((rural$ or remot$ or nonmetropolitan or non metropolitan or suburb$ or village$ or town$) adj3 (clinic$ or hospital$ or facilit$ or health$ cent$ or health care cent$? or health care cent$ or medical cent$)).mp. | 9511 |
| 11 | ((shortage$ or understaff$ or under staff$) adj3 area$).mp. | 229 |
| 12 | 2 or 3 or 4 or 5 or 6 or 7 or 8 or 9 or 10 or 11 | 1259850 |
| 13 | exp community health workers/ | 1534 |
| 14 | exp medical auxiliaries/ | 7261 |
| 15 | exp task shifting/ | 42 |
| 16 | exp health care workers/ | 110978 |
| 17 | (lay adj3 (worker? or visitor? or attendant? or aid$ or support$ or person$ or helper? or carer? or caregiver? or care giver? or counselor? or counsellor? or assistant? or staff)).mp. | 775 |
| 18 | ((voluntary or volunteer?) adj3 (worker? or visitor? or attendant? or aid$ or support$ or person$ or helper? or carer? or caregiver? or care giver? or consultant? or advisor? or counselor? or counsellor? or assistant? or staff)).mp. | 1602 |
| 19 | (untrained adj3 (worker? or visitor? or attendant? or aid$ or support$ or person$ or helper? or carer? or caregiver? or care giver? or consultant? or advisor? or counselor? or counsellor? or assistant? or staff or nurse? or doctor? or physician? or therapist?)).mp. | 284 |
| 20 | (trained adj3 (worker? or visitor? or attendant? or aid$ or support$ or person$ or helper? or carer? or caregiver? or care giver? or consultant? or advisor? or counselor? or counsellor? or assistant? or staff or nurse? or doctor? or physician? or therapist?)).mp. | 7495 |
| 21 | (unlicensed adj3 (worker? or visitor? or attendant? or aid$ or support$ or person$ or helper? or carer? or caregiver? or care giver? or consultant? or advisor? or counselor? or counsellor? or assistant? or staff or nurse? or doctor? or physician? or therapist?)).mp. | 25 |
| 22 | ((nonprofessional? or non professional?) adj3 (worker? or visitor? or attendant? or aid$ or support$ or person$ or helper? or carer? or caregiver? or care giver? or consultant? or advisor? or counselor? or counsellor? or assistant? or staff)).mp. | 95 |
| 23 | ((non medical or non health or non healthcare or non health care) adj3 (worker? or visitor? or attendant? or aid$ or support$ or person$ or helper? or carer? or caregiver? or care giver? or consultant? or advisor? or counselor? or counsellor? or assistant? or staff)).mp. | 483 |
| 24 | (community adj3 (worker? or visitor? or attendant? or aid$ or support$ or person$ or helper? or carer? or caregiver? or care giver? or consultant? or advisor? or counselor? or counsellor? or assistant? or staff)).mp. | 11893 |
| 25 | (paraprofessional? or paramedic or paramedics or paramedical worker? or paramedical personnel or allied health personnel or allied health worker? or support worker? or non specialist? or specially trained or barefoot doctor? or nurs$ aid$ or psychiatric aid$ or psychiatric attendant? or social worker? or teacher? or school staff or trainer?).mp. | 21865 |
| 26 | ((health$ or medical$) adj3 (auxiliary or auxiliaries)).mp. | 7525 |
| 27 | (nurs$ adj1 (auxiliary or auxiliaries)).mp. | 295 |
| 28 | (informal adj (caregiver? or care giver? or carer?)).mp. | 489 |
| 29 | (self help group? or support group?).mp. | 1670 |
| 30 | ((social or psychosocial) adj (care or support)).mp. | 13558 |
| 31 | (village adj3 worker?).mp. | 636 |
| 32 | community based.mp. | 28889 |
| 33 | (community adj3 intervention?).mp. | 5610 |
| 34 | community network?.mp. | 228 |
| 35 | ((health or health care or healthcare) adj manpower).mp. | 176 |
| 36 | human resources.mp. | 5071 |
| 37 | (task? adj3 shift$).mp. | 710 |
| 38 | (task? adj3 shar$).mp. | 284 |
| 39 | (staff$ adj3 chang$).mp. | 316 |
| 40 | 13 or 14 or 15 or 16 or 17 or 18 or 19 or 20 or 21 or 22 or 23 or 24 or 25 or 26 or 27 or 28 or 29 or 30 or 31 or 32 or 33 or 34 or 35 or 36 or 37 or 38 or 39 | 183968 |
| 41 | 1 and 12 and 40 | 491 |

CINAHL <1937 to Jan 22 2023>

| **#** | **Query** | **Results** |
| --- | --- | --- |
| S59 | S3 AND S20 AND S58 | 233 |
| S58 | S21 OR S22 OR S23 OR S24 OR S25 OR S26 OR S27 OR S28 OR S29 OR S30 OR S31 OR S32 OR S33 OR S34 OR S35 OR S36 OR S37 OR S38 OR S39 OR S40 OR S41 OR S42 OR S43 OR S44 OR S45 OR S46 OR S47 OR S48 OR S49 OR S50 OR S51 OR S52 OR S53 OR S54 OR S55 OR S56 OR S57 | 303,876 |
| S57 | (staff* N3 chang*) | 2,392 |
| S56 | ((task or tasks) N3 shift*) | 1,167 |
| S55 | ((task or tasks) N3 shar*) | 693 |
| S54 | ("human resources") | 22,824 |
| S53 | ((health or healthcare or "health care") W0 manpower) | 163 |
| S52 | ("community network*") | 2,955 |
| S51 | (community N3 intervention*) | 10,169 |
| S50 | "community based" | 41,047 |
| S49 | (village N3 worker*) | 193 |
| S48 | ((social or psychosocial) W0 (care or support)) | 48,123 |
| S47 | ("self help group" or "self help groups" or "support group" or "support groups") | 15,656 |
| S46 | (informal W0 (caregiver* or "care giver" or "care givers" or carer*)) | 4,210 |
| S45 | (nurs* N1 (auxiliary or auxiliaries)) | 584 |
| S44 | ((health* or medical*) N3 (auxiliary or auxiliaries)) | 164 |
| S43 | (paraprofessional* or paramedic or paramedics or paramedical W0 worker* or paramedical W0 personnel or "allied health personnel" or "allied health worker" or "allied health workers" or support W0 worker* or non W0 specialist* or "specially trained" or barefoot W0 doctor* or nurs* W0 aid* or psychiatric W0 aid* or psychiatric W0 attendant* or social W0 worker* or teacher* or "school staff" or trainer*) | 87,342 |
| S42 | (community N3 (worker* or visitor* or attendant* or aid* or support* or person* or helper* or carer* or caregiver* or "care giver" or "care givers" or consultant* or advisor* or counselor* or counsellor* or assistant* or staff)) | 27,483 |
| S41 | (("non medical" or "non health" or "non healthcare") N3 (worker* or visitor* or attendant* or aid* or support* or person* or helper* or carer* or caregiver* or "care giver" or "care givers" or consultant* or advisor* or counselor* or counsellor* or assistant* or staff)) | 510 |
| S40 | ((nonprofessional* or "non professional" or "non professionals") N3 (worker* or visitor* or attendant* or aid* or support* or person* or helper* or carer* or caregiver* or "care giver" or "care givers" or consultant* or advisor* or counselor* or counsellor* or assistant* or staff)) | 327 |
| S39 | (unlicensed N3 (worker* or visitor* or attendant* or aid* or support* or person* or helper* or carer* or caregiver* or "care giver" or "care givers" or consultant* or advisor* or counselor* or counsellor* or assistant* or staff or nurse* or doctor* or physician* or therapist*)) | 4,207 |
| S38 | (trained N3 (worker* or visitor* or attendant* or aid* or support* or person* or helper* or carer* or caregiver* or "care giver" or "care givers" or consultant* or advisor* or counselor* or counsellor* or assistant* or staff or nurse* or doctor* or physician* or therapist*)) | 12,792 |
| S37 | (untrained N3 (worker* or visitor* or attendant* or aid* or support* or person* or helper* or carer* or caregiver* or "care giver" or "care givers" or consultant* or advisor* or counselor* or counsellor* or assistant* or staff or nurse* or doctor* or physician* or therapist*)) | 369 |
| S36 | ((voluntary or volunteer*) N3 (worker* or visitor* or attendant* or aid* or support* or person* or helper* or carer* or caregiver* or "care giver" or "care givers" or consultant* or advisor* or counselor* or counsellor* or assistant* or staff)) | 17,869 |
| S35 | (lay N3 (worker* or visitor* or attendant* or aid* or support* or person* or helper* or carer* or caregiver* or "care giver" or "care givers" or consultant* or advisor* or counselor* or counsellor* or assistant* or staff)) | 1,635 |
| S34 | (MH "Community Mental Health Services") | 11,130 |
| S33 | (MH "Rural Health Personnel") | 758 |
| S32 | (MH "Allied Health Personnel") | 4,881 |
| S31 | (MH "Community Health Workers") | 4,360 |
| S30 | (MH "Nursing Assistants") | 12,648 |
| S29 | (MH "Nursing Assistants") | 12,648 |
| S28 | (MH "Volunteer Workers") | 15,590 |
| S27 | (MH "Community Networks") | 2,649 |
| S26 | (MH "Support Groups") | 11,411 |
| S25 | (MH "Health Manpower") | 1,059 |
| S24 | (MH "Health Manpower") | 1,059 |
| S23 | (MH "Personnel Staffing and Scheduling") | 23,318 |
| S22 | (MH "Home Health Aides") | 1,496 |
| S21 | (MH "Health Personnel, Unlicensed") | 3,980 |
| S20 | S4 OR S5 OR S6 OR S7 OR S8 OR S9 OR S10 OR S11 OR S12 OR S13 OR S14 OR S15 OR S16 OR S17 OR S18 OR S19 | 175,403 |
| S19 | ((shortage or understaffed or under W0 staffed) N3 (area or areas)) | 458 |
| S18 | ((rural* or remot* or nonmetropolitan or non W0 metropolitan or suburb* or village* or town*) N3 (clinic or clinics or hospital or hospitals or facility or facilities or health* W0 center or health* W0 centers or health* W0 centre or health* W0 centres or health care center or health care centers or health care centre or health care centres or medical center or medical centers or medical centre or medical centres)) | 30,035 |
| S17 | ((rural* or remot* or nonmetropolitan or non W0 metropolitan or suburb* or middle W0 income or low* W0 income or underserved or under W0 served or deprived or poor or village* or town*) N3 population* ) | 23,586 |
| S16 | ((rural* or remot* or nonmetropolitan or non W0 metropolitan or suburb* or developing or less* W0 developed or under W0 developed or underdeveloped or middle W0 income or low* W0 income or underserved or under W0 served or deprived or poor) N3 (communit* or area or areas or village* or town* or count* or region* or province* or setting*)) | 114,034 |
| S15 | (rural* W0 (health* or medical care or medical service*)) | 16,979 |
| S14 | (rural* or remot* or nonmetropolitan or non W0 metropolitan or suburb*) | 109,119 |
| S13 | (MH "Medically Underserved Area") | 2,642 |
| S12 | (MH "Medically Underserved") | 3,841 |
| S11 | (MH "Rural Areas") | 25,902 |
| S10 | (MH "Rural Health") | 7,484 |
| S9 | (MH "Rural Health") | 7,484 |
| S8 | (MH "Rural Health Services") | 7,471 |
| S7 | (MH "Rural Population") | 12,922 |
| S6 | (MH "Hospitals, Rural") | 3,246 |
| S5 | (MH "Rural Health Centers") | 416 |
| S4 | (MH "Rural Health Personnel") | 758 |
| S3 | S1 OR S2 | 22,395 |
| S2 | ((matern* or prenatal* or pre-natal* or antenatal* or ante-natal* or antepartum* or ante-partum* or perinatal* or peri-natal* or pregnan* or postpartum or post-partum or peripartum or peri-partum or postnatal* or post-natal* or puerper*) N3 (depress* or dysthymi* or MDD or distress or 'affective symptom*' or 'baby blues' or 'disorder*' or CMD or 'low mood*')) | 22,395 |

Cochrane CENTRAL

Search Name: CENTRAL_1.20

Date Run: 20/01/2023 16:22:48

Comment: 141 reviews, 21 protocols, 141 trials

| ID | Search | Hits |
| --- | --- | --- |
| #1 | MeSH descriptor: [Depression, Postpartum] explode all trees | 740 |
| #2 | ((matern* or prenatal* or pre-natal* or antenatal* or ante-natal* or antepartum* or ante-partum* or perinatal* or peri-natal* or pregnan* or postpartum or post-partum or peripartum or peri-partum or postnatal* or post-natal* or puerper*) NEAR/3 (depress* or dysthymi* or MDD or distress or (affective NEXT symptom*) or "baby blues" or disorder* or CMD or (low NEXT mood*))) | 5860 |
| #3 | #1 or #2 | 5860 |
| #4 | MeSH descriptor: [Rural Health Services] explode all trees | 362 |
| #5 | MeSH descriptor: [Rural Health] explode all trees | 545 |
| #6 | MeSH descriptor: [Suburban Health] explode all trees | 18 |
| #7 | MeSH descriptor: [Medically Underserved Area] explode all trees | 136 |
| #8 | MeSH descriptor: [Rural Population] explode all trees | 1969 |
| #9 | MeSH descriptor: [Hospitals, Rural] explode all trees | 38 |
| #10 | rural* or remot* or nonmetropolitan or (non NEXT metropolitan) or suburb* | 22037 |
| #11 | ((rural* NEXT health*) or (rural NEXT medical NEXT care) or (rural NEXT medical NEXT service*) or (rural NEXT medicine)) | 1463 |
| #12 | (rural* or remot* or nonmetropolitan or (non NEXT metropolitan) or suburb* or developing or (less* NEXT developed) or (under NEXT developed) or underdeveloped or (middle NEXT income) or (low* NEXT income) or underserved or (under NEXT served) or deprived or poor) NEAR/3 (communit* or area* or village* or town* or count* or region* or province* or setting*) | 17684 |
| #13 | (rural* or remot* or nonmetropolitan or (non NEXT metropolitan) or suburb* or (middle NEXT income) or (low* NEXT income) or underserved or (under NEXT served) or deprived or poor or village* or town* or count*) NEAR/3 population* | 5906 |
| #14 | (rural* or remot* or nonmetropolitan or (non NEXT metropolitan) or suburb* or village*) NEAR/3 (clinic* or hospital* or facilit* or (health* NEXT center*) or (health* NEXT centre*) or (health NEXT care NEXT center*) or (health NEXT care NEXT centre*) or (medical NEXT center*) or (medical NEXT centre*)) | 1829 |
| #15 | #4 or #5 or #6 or #7 or #8 or #9 or #10 or #11 or #12 or #13 or #14 | 33850 |
| #16 | MeSH descriptor: [Allied Health Personnel] this term only | 305 |
| #17 | MeSH descriptor: [Community Health Workers] this term only | 572 |
| #18 | MeSH descriptor: [Nursing Assistants] this term only | 71 |
| #19 | MeSH descriptor: [Psychiatric Aides] this term only | 3 |
| #20 | MeSH descriptor: [Caregivers] this term only | 2633 |
| #21 | MeSH descriptor: [Volunteers] this term only | 333 |
| #22 | MeSH descriptor: [Community Networks] this term only | 175 |
| #23 | MeSH descriptor: [Self-Help Groups] explode all trees | 801 |
| #24 | MeSH descriptor: [Social Support] this term only | 3473 |
| #25 | (lay NEAR/3 (worker* or visitor* or attendant* or aid* or support* or person* or helper* or carer* or caregiver* or "care giver*" or "care givers" or consultant* or advisor* or counselor* or counsellor* or assistant* or staff)) | 916 |
| #26 | ((voluntary or volunteer*) NEAR/3 (worker* or visitor* or attendant* or aid* or support* or person* or helper* or carer* or caregiver* or "care giver*" or "care givers" or consultant* or advisor* or counselor* or counsellor* or assistant* or staff)) | 1277 |
| #27 | (untrained NEAR/3 (worker* or visitor* or attendant* or aid* or support* or person* or helper* or carer* or caregiver* or "care giver*" or "care givers" or consultant* or advisor* or counselor* or counsellor* or assistant* or staff or nurse* or doctor* or physician* or therapist*)) | 132 |
| #28 | (trained NEAR/3 (worker* or visitor* or attendant* or aid* or support* or person* or helper* or carer* or caregiver* or "care giver*" or "care givers" or consultant* or advisor* or counselor* or counsellor* or assistant* or staff or nurse* or doctor* or physician* or therapist*)) | 7437 |
| #29 | (unlicensed NEAR/3 (worker* or visitor* or attendant* or aid* or support* or person* or helper* or carer* or caregiver* or "care giver*" or "care givers" or consultant* or advisor* or counselor* or counsellor* or assistant* or staff or nurse* or doctor* or physician* or therapist*)) | 25 |
| #30 | ((nonprofessional* or "non professional*" or "non professionals") NEAR/3 (worker* or visitor* or attendant* or aid* or support* or person* or helper* or carer* or caregiver* or "care giver" "care givers" or "care givers" or consultant* or advisor* or counselor* or counsellor* or assistant* or staff)) | 123 |
| #31 | (("non medical" or "non health" or "non healthcare" or "non health care") NEAR/3 (worker* or visitor* or attendant* or aid* or support* or person* or helper* or carer* or caregiver* or "care giver*" "care givers" or consultant* or advisor* or counselor* or counsellor* or assistant* or staff)) | 119 |
| #32 | ((community or village) NEAR/3 (worker* or visitor* or attendant* or aid* or support* or person* or helper* or carer* or caregiver* or "care giver*" or "care givers" or consultant* or advisor* or counselor* or counsellor* or assistant* or staff)) | 6530 |
|  |  |  |
| #33 | (paraprofessional* or paramedic or paramedics or "paramedical worker" or "paramedical workers" or "paramedical personnel" or "allied health personnel" or "allied health worker" or "allied health workers" or (support NEXT worker*) or ("non NEXT specialist*") or ("non NEXT specialists") or "specially trained" or (barefoot NEXT doctor*) or (nurse* NEXT aid*) or (psychiatric NEXT aid*) or (psychiatric NEXT attendant*) or (social NEXT worker*) or teacher* or "school staff" or trainer*) | 14660 |
| #34 | ((health* or medical*) NEAR/3 (auxiliar*)) | 771 |
| #35 | (nurs* NEAR/1 (auxiliar*)) | 80 |
| #36 | (informal NEXT (caregiver* or "care giver*" or "care givers" or carer*)) | 955 |
| #37 | ("self help group" or "self help groups" or "support group" or "support groups") | 3240 |
| #38 | ((social or psychosocial) NEXT (care or support)) | 13252 |
| #39 | "community based" | 11525 |
| #40 | (community NEAR/3 intervention*) | 5310 |
| #41 | ("community network" or "community networks") | 251 |
| #42 | ((health or "health care" or healthcare) NEXT manpower) | 24 |
| #43 | "human resources" | 415 |
| #44 | (task* NEAR/3 shift* or taskshift*) | 488 |
| #45 | (task* NEAR/3 shar* or taskshar*) | 147 |
| #46 | #16 or #17 or #18 or #19 or #20 or #21 or #22 or #23 or #24 or #25 or #26 or #27 or #28 or #29 or #30 or #31 or #32 or #33 or #34 or #35 or #36 or #37 or #38 or #39 or #40 or #41 or #42 or #43 or #44 or #45 | 55740 |
| #47 | #3 and #15 and #46 | 282 |

Global Index Medicus

| (tw:(tw:((tw:(tw:(tw:((tw:(( mh:("Depression, Postpartum")))) OR (tw:(((dysthmi* OR depress* OR disorder* OR mdd OR cmd) AND (mother* OR matern* OR pregnan* OR postpartum* OR antepartum* OR peripartum* OR prenatal* OR antenatal* OR postnatal* OR perinatal*) ))))))) AND (tw:((rural* OR remot* OR villag* OR town* OR countr* OR nonmetro* OR suburb OR understaff* OR shortage*)))))) AND tw:(lay OR community or volunteer* or voluntary or untrained or trained or nonprofessional* or “non professional” or “non professionals” or “non specialist” or “non specialists” or unlicensed or paraprofessional or “allied health” or “barefoot doctor” or “barefoot doctors” or aid* or worker* or auxiliar* or informal or shar* or shift*) | 931 |
| --- | --- |

- Filter: English only > 489

##

**Table E** Contacted Experts

Experts in the field of maternal mental health who were contacted to ensure this review was as comprehensive as possible include the following:

| **Name** | **Associated Institution** |
| --- | --- |
| Simone Honikman, MD | Perinatal Mental Health Project, South Africa |
| Cindy Lee Dennis, PhD | University of Toronto, Canada |
| Jane Fisher, PhD | Monash University, Australia |
| Tatiana Salisbury, PhD | King’s College London, UK |
| Alan Stein, MD | University of Oxford, UK |

1. Participants with co-occurring mental health conditions (e.g., anxiety, substance use, etc) will be included [↑](#footnote-ref-1)
2. There is no predetermined cutoff point for categorization (PD vs not) used with screening tools such as the EPDS [↑](#footnote-ref-2)
3. Pregnancy or within 12 months of delivery [↑](#footnote-ref-3)
4. Congestive heart failure, cancer, etc. [↑](#footnote-ref-4)
5. No limitation on location of delivery, method of delivery (e.g., video chat, phone, in-person), group vs. individual [↑](#footnote-ref-5)
6. “Non-specialist” includes anyone without specialized mental health training (e.g., primary care physicians, nurses, midwives, lay persons, health workers/volunteers, etc.) [↑](#footnote-ref-6)
7. Intervention itself does NOT need to be specifically designed for perinatal depression as long as it measures remission or symptom severity as an outcome [↑](#footnote-ref-7)
8. E.g., psychiatrist, psychologist, or trained/qualified mental health professionals [↑](#footnote-ref-8)
9. E.g., general parenting education such as prenatal or infant care classes [↑](#footnote-ref-9)
10. For studies that include participants in both rural and urban locations but do not report segregated results for the rural sub-sample, reviewers will try to contact study authors [↑](#footnote-ref-10)
